# Triiodothyronine modulates neuronal plasticity mechanisms to enhance functional outcome after stroke

**DOI:** 10.1186/s40478-019-0866-4

**Published:** 2019-12-21

**Authors:** Daniela Talhada, Joana Feiteiro, Ana Raquel Costa, Tiago Talhada, Elisa Cairrão, Tadeusz Wieloch, Elisabet Englund, Cecília Reis Santos, Isabel Gonçalves, Karsten Ruscher

**Affiliations:** 1 Laboratory for Experimental Brain Research, Division of Neurosurgery, Department of Clinical Sciences, Lund University, BMC A13, S-22184 Lund, Sweden; 2 CICS-UBI-Health Sciences Research Centre, Faculdade de Ciências da Saúde, Universidade da Beira Interior, Covilhã, Portugal; 3 Division of Oncology and Pathology, Lund University Hospital, Lund, Sweden; 4 LUBIN Lab - Lunds Laboratorium för Neurokirurgisk Hjärnskadeforskning, Division of Neurosurgery, Department of Clinical Sciences, Lund University, Lund, Sweden

**Keywords:** Ischemia, Photothrombosis, Recovery, Stroke, Thyroid hormones, Thyroid hormone receptors, 3,5,3′-triiodo-L-thyronine (T_3_), 3,5,3′,5′-tetraiodo-L-thyronine (T_4_)

## Abstract

The development of new therapeutic approaches for stroke patients requires a detailed understanding of the mechanisms that enhance recovery of lost neurological functions. The efficacy to enhance homeostatic mechanisms during the first weeks after stroke will influence functional outcome. Thyroid hormones (TH) are essential regulators of neuronal plasticity, however, their role in recovery related mechanisms of neuronal plasticity after stroke remains unknown. This study addresses important findings of 3,5,3′-triiodo-L-thyronine (T_3_) in the regulation of homeostatic mechanisms that adjust excitability – inhibition ratio in the post-ischemic brain. This is valid during the first 2 weeks after experimental stroke induced by photothrombosis (PT) and in cultured neurons subjected to an in vitro model of acute cerebral ischemia. In the human post-stroke brain, we assessed the expression pattern of TH receptors (TR) protein levels, important for mediating T_3_ actions.

Our results show that T_3_ modulates several plasticity mechanisms that may operate on different temporal and spatial scales as compensatory mechanisms to assure appropriate synaptic neurotransmission. We have shown in vivo that long-term administration of T_3_ after PT significantly (1) enhances lost sensorimotor function; (2) increases levels of synaptotagmin 1&2 and levels of the post-synaptic GluR2 subunit in AMPA receptors in the peri-infarct area; (3) increases dendritic spine density in the peri-infarct and contralateral region and (4) decreases tonic GABAergic signaling in the peri-infarct area by a reduced number of parvalbumin^+^ / c-fos^+^ neurons and glutamic acid decarboxylase 65/67 levels. In addition, we have shown that T_3_ modulates in vitro neuron membrane properties with the balance of inward glutamate ligand-gated channels currents and decreases synaptotagmin levels in conditions of deprived oxygen and glucose. Interestingly, we found increased levels of TRβ1 in the infarct core of *post-mortem* human stroke patients, which mediate T_3_ actions. Summarizing, our data identify T_3_ as a potential key therapeutic agent to enhance recovery of lost neurological functions after ischemic stroke.

## Introduction

Loss of motor function following ischemic stroke is the most enduring and disabling consequence [4, 26]. Despite the attempt to find neuroprotective treatments that mitigate tissue damage and loss of motor function, their translation into clinical practice has been disappointing. So far, thrombectomy and thrombolysis in the acute phase after stroke are the only effective treatments to restore blood flow and minimize brain damage. However, acute therapies are limited to the first 4.5 h for thrombolysis or up to 24 h for thrombectomy after stroke onset and are accessible to less than 10% of stroke patients [36, 49]. Beyond the acute phase constant and consistent specific rehabilitation programs are instrumental to partially regain brain function, dependent on size and brain regions affected by stroke [37]. Therefore, the options to minimize the damage after ischemic stroke remains sub-optimal and there is need for new therapeutic approaches that target restorative processes.

In response to loss of input from the infarct core, surviving neurons adopt self-repair and self-organizing homeostatic mechanisms in order to stabilize the ratio between excitatory and inhibitory circuits and maintain adequate synaptic input [21]. First, blood flow is restored and there is a temporary resolution in neuronal activity and metabolism in regions surrounding and connected to the infarct [33, 82]. Concomitantly, in response to cell death in the infarct core, there is a change in neuronal pathways and reorganization of neuronal connectivity, namely axonal growth, spine remodeling and dendritic arborization [29, 82]. A wide variety of homeostatic mechanisms contribute to the maintenance of overall excitability, involving the regulation of neuronal intrinsic excitability and synaptic transmission. These mechanisms include changes in receptor expression at the post-synaptic level, neurotransmitter release at the pre-synaptic level, ion channel function and synapse number or synaptic strength [52, 76, 77].

Processes of neuronal reorganization and cellular responses to the infarct occur during the first weeks after stroke in mice [7] and up to months and years in humans [23, 24]. During this period the brain is highly plastic, and distinct overlapping events promoting recovery of neurological function can be modulated by external interventions [82]. Therefore, current research is focused to understand mechanisms of post-injury plasticity that occur spontaneously after stroke [50, 82].

Current epidemiological human studies suggest that thyroid hormones (TH) signaling is related to a better outcome after stroke although the mechanisms involved are poorly investigated [71]. Several studies have pointed out that TH contribute to neuroprotection when administered before [46, 61] or during the first hours after stroke or traumatic brain injury [20, 22, 27, 40, 55]. TH also protect cortical neurons against glutamate-induced neuronal damage [42].

However, long-term effects of TH during the recovery phase after stroke remain largely unknown [71]. Here we hypothesized that 3,5,3′,5′-tetraiodo-L-thyronine (T_4_) and 3,5,3′-triiodo-L-thyronine (T_3_) might be actively involved to enhance post-stroke recovery, since they are essential in several mechanisms for brain development [3, 5] and normal function of the adult brain [47]. Summarizing, TH are involved in mechanisms of neuronal proliferation, migration and differentiation, neurite outgrowth, synaptic plasticity, dendritic branching and myelination during brain development [5, 28]. In the adult brain, several processes of neurorepair are particularly dependent on T_3_ action, namely neuronal plasticity and neurogenesis [35, 56].

To study the role of TH in mechanisms of neuronal repair, we analyzed post-ischemic brains of mice subjected to intraperitoneal (i.p.) administration of T_4_ and T_3_ at 5 or 50 μg/kg starting at day two after photothrombosis (PT) and every second day, in a total of six administrations; we assessed T_3_ effects in ionotropic glutamate receptors (iGluRs) in cultured glutamatergic neurons; and we analyzed expression pattern of TH receptors (TR) alpha 1 (TRα1) and beta 1 (TRβ1) in post-ischemic brains of mice and human patients. In the present investigation we demonstrate that T_3_ modulates pathways during critical periods of recovery after stroke involved in reorganization of neuronal circuits and synaptic plasticity, functional connectivity and motor recovery. Summarizing, we demonstrate that (1) T_3_ enhanced recovery of lost motor function in an experimental model of stroke, (2) T_3_ increased levels of synaptotagmin 1&2 and levels of post-synaptic glutamate receptor 2 (GluR2) subunit in alpha-amino-3-hydroxy-5-methyl-4-isoxazolepropionic acid (AMPA) receptors in the peri-infarct area, (3) T_3_ increased dendritic spine density in the ipsilateral and contralateral regions and (4) T_3_ decreased tonic GABAergic signaling in the peri-infarct area by a reduced number of parvalbumin-positive (PV^+^) / c-fos^+^ neurons and glutamic acid decarboxylase 65/67 (GAD 65/67) protein levels. In cultured neurons (5) T_3_ modulates membrane properties with the balance of inward glutamate ligand-gated channels currents and (6) T_3_ modulates synaptotagmin levels in an in vitro model of ischemia. In the human post-ischemic brain (7) TRβ1 has a spatial expression pattern, which may drive T_3_ transcriptional activity.

## Materials and methods

### Ethical considerations

Mice were bred and genotyped at the conventional facility of the Biomedical Centre, (BMC, Lund, Sweden). All animal experiments (*Studies I and II*) were carried out in accordance with the international guidelines on experimental animal research, with the approval of the Malmö-Lund Ethical Committee (ethical permit no. M50/2015) and followed the ARRIVE guidelines. All in vitro experiments (*Study III*) were carried out in compliance with directives on animal experimentation (Decreto-Lei 113/2013 and 2010/63/EU) in Portugal and European Union and with approval of the committee of Animal Research at Universidade da Beira Interior (CICS-UBI, Covilhã, Portugal). Human brain tissue used in this study was used with the approval of the Lund Ethical Review Board for research involving humans (Dnr 2011/80).

### Thyroid hormones effects after experimental stroke (study I)

**Fig. 1:**
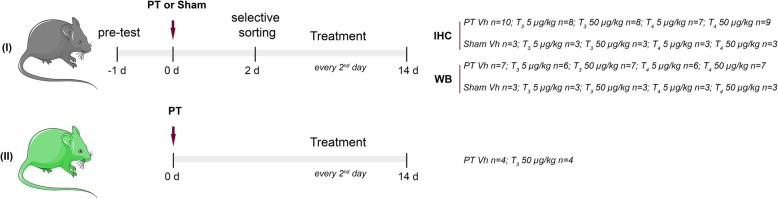
Experimental design. In *Study I* C57BL/6 mice were pre-tested before photothrombosis (PT) or sham operations to assess limb placement. Selective sorting was assessed 2 days after surgeries. Animals were randomized into the treatment groups: Vehicle (Vh, NaCl 0.9%); T_3_ 5 or 50 μg/kg; T_4_ 5 or 50 μg/kg. Treatment was administrated via intraperitoneal injection every second day after PT or sham operations. Neurological outcome was assessed by the rotating pole test, seven and 14 days after surgeries and brains were perfusion fixed or frozen, for immunohistochemistry (IHC) or Western blot (WB), respectively. *Study II* was performed for dendritic spine analysis and Thy1-YFP transgenic mice were used. Treatment with Vh or T_3_ 50 μg/kg was administered as described for *Study I*

For this study, 117 C57BL/6 male mice (20 to 26 g, aged 9 to 10 weeks, purchased from Charles River) were used. Out of 117 animals, 12 were excluded due to problems during surgery and mortality before entering the treatment phase and 105 animals were randomly assigned into the treatment groups (Fig. 1). Treatment was initiated on day two after PT and every other day until the endpoint of the study. Vehicle (Vh, NaCl 0.9%), T_3_ (5 or 50 μg/kg) or T_4_ (5 or 50 μg/kg) were administered by i.p. injection in a total of six administrations. On days two, seven and 14 after stroke onset or sham surgery, animals were evaluated for motor function.

#### Photothrombosis

Focal ischemic stroke was induced by PT, as described previously [65, 81]. Ischemic stroke was induced in the right hemisphere through illumination of a squared aperture measuring 4.0 to 2.0 mm (equal to an area of 8.0 mm^2^). The light position related to bregma (+ 1.5 mm lateral and + 0.5 mm anterior) affected the mouse primary motor cortex of forelimb-responsive sites, in the left body side [72]. The same procedure was performed in sham operated animals, with saline injection instead of photosensitizing dye.

#### Behavior analysis

Motor function and exploratory behavior after TH treatment was assessed using a neuroscore consisting of the rotating pole test (RPT) and the open field test, respectively [60, 79]. These assessments were performed in a blinded fashion to the investigator that performed the surgeries and treatments.

The RPT was used to assess postural and locomotor asymmetry that results from an unilateral brain lesion [57]. After stroke or sham surgery, animals were evaluated on day two for randomization into treatment groups. Each trial was video recorded, and videos were used to assess motor dysfunction by using a zero to six scoring system (Table 1). Animals that did not fulfill inclusion criteria were excluded from behavior analysis (see Additional file 1: Supplementary methods).

**Table 1:** Motor function assessed by the rotating pole test before and after photothrombosis at days 2, 7 and 14

Score	Criteria
0	animal falls off immediately upon entry onto the pole
1	animal remains embraced to the pole unable to cross and eventually falls off the pole
2	animal falls off during crossing or if the hindlimbs do not contribute to forward movement
3	animal crosses the pole while continuously slipping with the forelimbs or hindlimbs
4	animal crosses pole with > 3 ft slips
5	animal traverses the pole with 1–3 ft slips
6	animal crosses the pole without any foot slips

The open field test was performed 14 days after stroke to assess both spontaneous post-ischemic locomotor activity and post-ischemic exploration behavior [78].

#### Immunohistochemistry and immunofluorescence

Tissue collection for immunostainings was performed as described before [53, 63]. Primary antibodies used for immunofluorescence were rabbit TRβ1 (Millipore, 1:1000), rabbit TRα1 (Abcam, 1:1000), goat parvalbumin (PV235, Swant, 1:5000), mouse neuronal nuclei (NeuN, Millipore, 1:1000), glial fibrillary acidic protein (GFAP)-Cy3 (Sigma, 1:5000), rat cluster of differentiation (CD) 68 (Abd Serotec, 1:300), and mouse glutathione S-transferase (GST)-pi isoform (BD Transduction Laboratories, 1:1000).

#### Infarct size measurement

Coronal brain sections from the start until the end of the infarct and spaced one millimeter were collected and stained for NeuN (rabbit NeuN, Millipore, 1:5000). The non-injured portion of the ipsilateral and contralateral hemisphere were encircled and the indirect infarct volume was calculated by integration of areas from serial sections of each brain as described previously [70] using Fiji software [64].

#### Counting of parvalbumin positive cells

For each animal one coronal section (− 2.0 mm relative to bregma) was stained for PV^+^ neurons using a monoclonal goat primary antibody (PV235, Swant, 1:5000), and visualization accessed using a VECTOR NovaRED Peroxidase (HRP) Substrate Kit (Vector Laboratories, CA, USA). Rabbit c-fos (Santa Cruz, 1:500) positive immunoreactivity (c-fos^+^) was accessed using the avidin–biotin–HRP system.

#### Immunoblotting

Brains from mice were collected as previously described [63] and the tissue correspondent to the infarct core and peri-infarct was collected. Tissue from human brains were dissected out by a pathologist following autopsy. Primary antibodies used for Western blots were rabbit TRα1 (Abcam, 1:1000), rabbit TRβ1 (Millipore, 1:20000), mouse postsynaptic protein 95 (PSD95; BD Transduction Laboratories, 1:1000), rabbit synaptophysin (Thermoscientific, 1:15000), rabbit GluR1 (Millipore, 1:2000), mouse GluR2 (Millipore, 1:1000), mouse *N*-methyl-D-aspartate receptor 1 (NMDAR1) (BD Transduction Laboratories, 1:1000), rabbit synaptotagmin 1&2 (Abcam, 1:1000) and rabbit GAD 65/67 (Millipore, 1:2000). Membranes were reprobed with anti β-actin HRP conjugated (1:150000, Sigma-Aldrich). Levels were calculated as a percentage of β-actin expression, after densitometric analysis using Fiji software.

### Dynamics of dendritic spines after administration with T_3_ (study II)

To study the effects of T_3_ on dendritic spine dynamics in mouse neocortical neurons after experimental stroke, eight Thy1-yellow fluorescent protein (YFP) transgenic mice (25 to 40 g, aged 1 year, own breeding), that express YFP in neuronal population were used. Mice were randomly assigned in the following treatment groups: PT/Vh, *n* = 4; PT/T_3_ 50 μg/kg, *n* = 4 (Fig. 1). Treatment was administered as described above for *Study I*. Fourteen days after the surgery, mice were sacrificed, perfusion fixed with paraformaldehyde 4% and brains were collected for further infarct volume assessment and dendritic spine analysis.

#### Photothrombosis

To induce PT in animals for dendritic spine analysis (*Study II*) the surgical procedure was performed as in *Study I*, and the left hemisphere was illuminated with a cold light source through a round aperture measuring 1.5 mm in diameter (equal to an area of 1.767 mm^2^) for 20 min. This approach induced smaller infarct sizes so that dendritic spines could be analyzed in different regions in the peri-infarct area. The same procedure was performed in Sham operated animals, with saline injection instead of photosensitizing dye.

#### Detection and classification of dendritic spines from fluorescence laser scanning microscopy

Three coronal sections per animal were collected at different levels: + 2.0 mm, + 1.0 mm and 0 mm relatively to bregma, corresponding to the rostral pole, center and caudal pole of the infarct, respectively. For each animal, we analyzed layers II/III correspondent to the apical pyramidal neurons in the ipsilateral motor cortex (Region 1, R1), ipsilateral somatosensory cortex (Region 2, R2), contralateral motor cortex (Region 3, R3) and contralateral somatosensory cortex (Region 4, R4).

Dendritic spine density and shape classification was accurately quantified and characterized using a three-dimensional computational approach as previously described, after image deconvolution [58].

For each region, three to five dendritic branches were randomly selected. Dendrites were manually selected, and spines were automatically detected using NeuronStudio software. Dendritic spines were classified according to the head to neck ratio and head diameter as stubby, mushroom or thin [30, 58], using default parameters from NeuronStudio. Dendritic spine density was calculated with the ratio number of spines / dendrite length.

### In vitro modulation of T_3_ in glutamatergic neurons (study III)

An in vitro model of cerebral ischemia and electrophysiology studies were performed to study immediate effects of T_3_ in homeostatic plastic mechanisms, namely modulation of synaptic proteins crucial for neurotransmission and NMDA and AMPA evoked currents.

#### Cell cultures

*C*ultured cortical neurons were used after 7–8 days in vitro (DIV). Primary cortical neuronal cultures were prepared as described before [59]. Cells were obtained from the cerebral cortex from Wistar rats on embryonic day 16–18. Briefly, meninges were removed, and the cortex dissected and subjected to enzymatic dissociation, using 0.05 / 0.02% w/v in phosphate buffered saline (PBS) trypsin / EDTA (#15400054, Thermofisher) for 15 min at 37 °C. The homogenized was rinsed with Dulbecco’s Modified Eagle’s medium (#11880036, DMEM, GIBCO) with 10% fetal bovine serum (#10500–064, GIBCO), 100 U penicillin and streptomycin/ml (#15140122, Thermofisher), 2 mM L-glutamine (#G5792, Sigma-Aldrich), dissociated with a Pasteur pipette, centrifuged and redissociated in starter medium (#21103049, Neurobasal medium, GIBCO) supplemented with B27 (#17504044, GIBCO), 100 U penicillin and streptomycin/ml, 2 mM L-glutamine (#G5792, Sigma-Aldrich) and 25 μM glutamate (#49621, Sigma-Aldrich). The cells were plated onto poly-L-lysine (#P4707, Sigma-Aldrich) pre-coated multiwells at 1.5 × 10^5^ cells/cm^2^ and grown in starter medium at 37 °C and 5% CO_2_. One-half of the medium was replaced with cultivating medium (starter medium without glutamate) from 4 DIV. Cells were used after 7–8 DIV for in vitro assays.

#### In vitro ischemic model and experimental treatments

After 7 DIV neurobasal medium was collected and stored to be replaced after the experiments. Neuron cultures were washed with PBS, and oxygen and glucose deprivation (OGD) was induced with a deoxygenated aglycemic solution. OGD was generated in a hypoxia incubator chamber (StemCell Technologies), flushed with gas: 5% CO_2_, 95% N_2_. In control cultures, medium was replaced by basic salt solution (BSS) after washing with PBS and cells were incubated in a normoxic atmosphere containing 5% CO_2_. Cultures were in OGD or BSS solutions for 120 min and after replaced by the previous collected medium. After OGD / BSS conditions, cells were incubated with Vh (DMSO in PBS, 0.01%) or T_3_ 1 μM for 48 h. Subsequently, cells were washed with cold PBS to remove excess of culture medium and cells collected and frozen at − 80 °C until protein extraction.

#### Immunocytochemistry

For immunocytochemistry, neurons were plated on glass coverslips and fixed after 7 DIV. Antibodies used for immunofluorescence were rabbit TRα1 (Thermoscientific, 1:500) or rabbit TRβ1 (Millipore, 1.500). The next day, neurons were stained with Hoechst-33,342 (4 μg/ml, Life Technologies).

#### Immunobloting

Protein extraction was performed as previously described [38, 63]. Western blot was performed to evaluate levels of mouse synaptotagmin (BD Transduction Laboratories, 1:2000).

#### Electrophysiological recording of membrane currents

To study ligand-gated channels AMPA and NMDA, we adopted the voltage-ramp method [85].

Individual currents were recorded after incubation with T_3_ 1 μM (*n* = 4) or Vh (*n* = 3) during the 48 h preceding the experiments. A sequence of voltage ramps at a rate of 0.23 mV/millisecond were applied at a holding potential of − 80 mV. To obtain the agonist induced current-voltage (I-V) relation, ramps I-V curves were constructed applying a 500 milliseconds voltage ramp ranging from − 110 mV to + 20 mV elicited every 8 s. Voltage ramps were applied in the absence and in the presence of AMPA and NMDA agonist glutamate at 50 μM and co-agonist of NMDA channels glycine at 3 μM, to enable subtraction of leak currents. The antagonists of AMPA and NMDA channels, 6-cyano-7-nitroquinoxaline-2,3-dione (CNQX; Sigma-Aldrich) and dizocilpinehydrogen maleate (MK-801; Sigma-Aldrich), respectively, were used both at 10 μM.

Cell currents were recorded sequentially in the presence of specific K^+^- channel blockers tetraethylammonium sodium salt (5 mM) and 4-Aminopyridine (1 mM), that were applied in the perfusion system together with the other drugs. Voltage-gated K^+^ channels needed to be blocked, since those channels were contributing to the conductance as well to the reversal potential obtained.

### Statistical analysis

Data are expressed as means ± standard error of the mean (SEM) for parametric data or as medians for non-parametric data. *P* values < 0.05 were considered as statistically significant. Statistical analysis was performed using IBM SPSS statistics 24 software for dendritic spine analysis or GraphPad Prism 6.0 software (GraphPad, San Diego, CA, USA), using one-way analysis of variance (ANOVA) followed by Bonferroni’s multiple comparison test when three or more groups were present or two-tailed unpaired Student’s *t*-test when comparing two groups. For non-parametric data, Kruskal Wallis test was employed for more than two groups followed by the Dunn’s multiple comparisons test and the Mann-Whitney *U*-test for comparison of two groups. Graphs were designed using GraphPad Prism 6.0 software.

For additional details about techniques and analysis performed, please refer to the Additional file 1: Supplementary Methods.

## Results

### Treatment with T_3_ improves functional recovery after PT without affecting infarct size

**Fig. 2:**
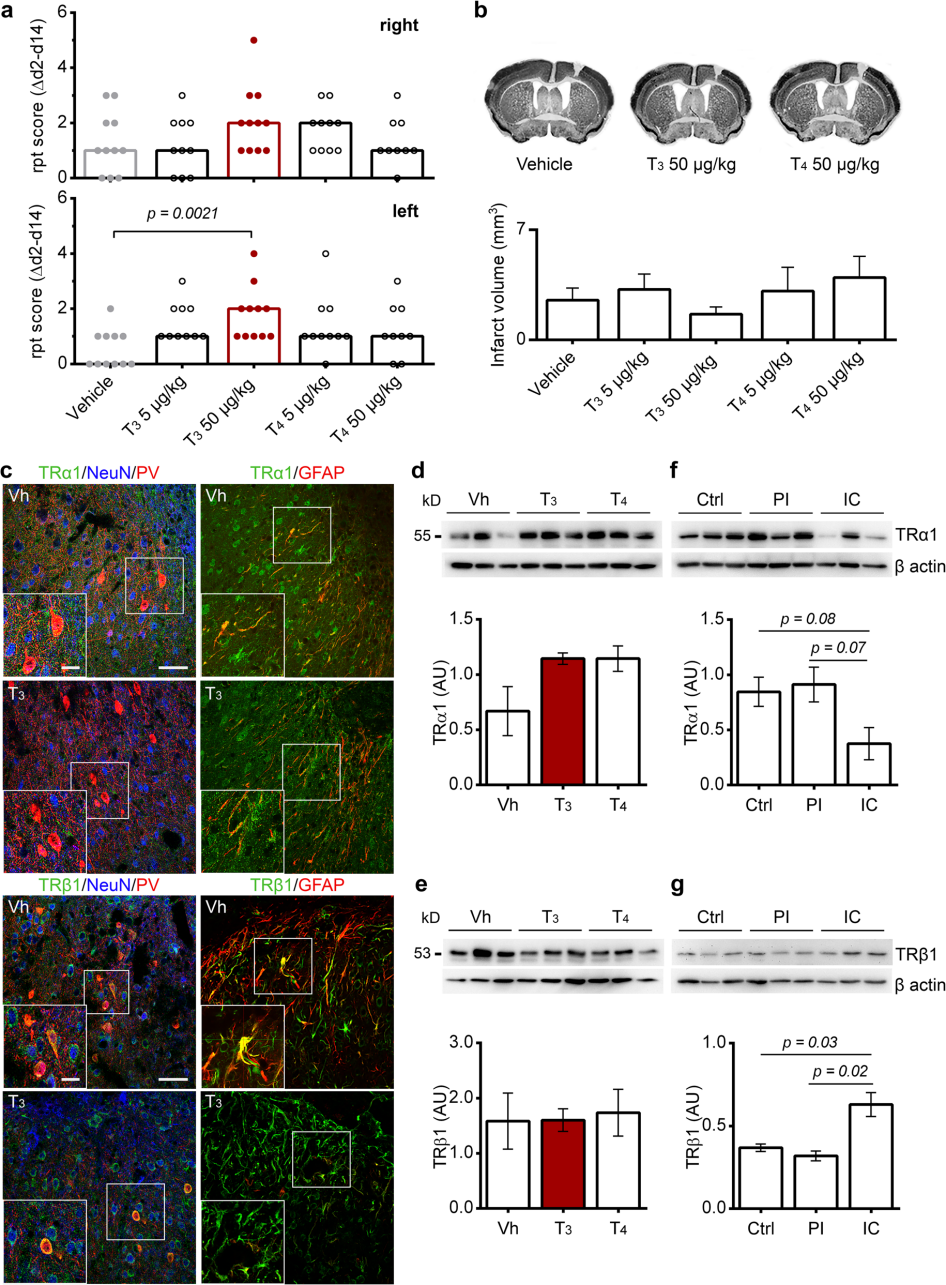
Treatment with T_3_ 50 μg/kg improves functional recovery 14 days after photothrombosis (PT) without affecting infarct size. **a** Difference between the rotating pole test (rpt) scores from day 2 (selective sorting) and 14 (Δd2-d14) at 10 rotations per minute to the right and to the left sides, from mice subjected to PT (right hemisphere). Scores are shown as individual data and group median. Statistical analysis was performed by Kruskal-Wallis test followed by Mann-Whitney test (*p* = 0.0021 in T_3_ 50 μg/kg versus Vehicle treatment). Vehicle (*n* = 11), T_3_ 5 μg/kg (*n* = 10), T_3_ 50 μg/kg (*n* = 11), T_4_ 5 μg/kg (*n* = 10), T_4_ 50 μg/kg (*n* = 9). **b** Representative coronal brain sections from stroke mice treated with Vehicle, T_3_ 50 μg/kg or T_4_ 50 μg/kg. Staining with NeuN was performed to measure cortical infarcts. Infarct volumes are displayed as means ± SEM. On day 14 after PT in mice treated with Vehicle (*n* = 10), T_3_ 5 μg/kg (*n* = 8), T_3_ 50 μg/kg (*n* = 8), T_4_ 5 μg/kg (*n* = 7) or T_4_ 50 μg/kg (*n* = 7). Statistical analysis was performed by one-way ANOVA and Bonferroni’s multiple comparisons test. **c** Thyroid hormone receptors (TR) α1 and TRβ1 (AF488, green) expression in mouse brain cell populations. Both TR isoforms are expressed in NeuN (Cy5, blue) positive neurons and Parvalbumin (Cy3, red) positive neurons. GFAP (Cy3, red) immunoreactive astrocytes express TRβ1 in the ischemic territory, 14 days after PT. Scale bars 50 μm and 10 μm for insets at higher magnification. **d** Levels of TRα1 and **e** TRβ1 in the infarct core and peri-infarct area were analyzed 14 days after PT and after treatment with Vehicle (*n* = 3), T_3_ 50 μg/kg (*n* = 3) or T_4_ 50 μg/kg (*n* = 3). No difference was observed in levels of TRα1 and TRβ1. **f** Levels of TRα1 and **g** TRβ1 in the grey matter of human brain in non-stroke (Ctrl), and stroke cases, including the peri-infarct (PI) and infarct core (IC). For uncropped images of western blots see Additional file 1: Figure S5. Levels of TRβ1 are increased in the IC. Statistical analysis was performed by One-way ANOVA and Bonferroni’s multiple comparisons test. Two-tailed unpaired Student’s *t* test was employed to determine *p* values. Data are expressed as mean ± SEM


We first assessed if treatment with T_3_ or T_4_ at 5 or 50 μg/kg enhances motor function in mice subjected to unilateral PT. Motor function was assessed by RPT on day 7 and 14 after stroke onset. We observed some degree of spontaneous recovery in mice of all groups subjected to PT. T_3_-treated mice at 50 μg/kg could traverse the pole with a score higher than three at 10 rpm, to the right and left sides, showing that all animals crossed the pole without falling (Additional file 2: Video S1, Additional file 3: Video S2, Additional file 4: Video S3 and Additional file 5: Video S4). However, a significantly enhanced functional recovery was only observed when the pole rotated at 10 rpm to the left, in animals treated with T_3_ at 50 μg/kg, when compared to Vh-treated animals (Fig. 2a). Fourteen days after stroke, 73% (eight out of 11) and 64% (seven out of 11) of mice treated with T_3_ 50 μg/kg had a score higher or equal to four points, at 3 rpm and 10 rpm to the left, respectively. In contrast, only 9% (one out of 11) of mice subjected to PT and treated with saline had a score of four points and not higher, at 3 and 10 rpm to the left (Additional file 1: Figure S1).

**Additional file 2.** Rotating pole test mouse 1 selective sorting after photothrombosis.



**Additional file 3.** Rotating pole test mouse 1 after vehicle treatment at 14 days.



**Additional file 4.** Rotating pole test mouse 2 selective sorting after photothrombosis.



**Additional file 5.** Rotating pole test mouse 2 after T_3_ treatment (50 µg/kg) at 14 days.


Infarct size influences the severity of neurological deficits and differences of infarct size among treatment groups may influence behavior assessment to evaluate motor recovery over time. Overall the infarct volume did not differ between animals assigned to treatment groups (2.5 ± 0.78 mm^3^ Vh, 3.2 ± 0.97 mm^3^ T_3_ 5 μg/kg, 1.6 ± 0.47 mm^3^ T_3_ 50 μg/kg, 3.1 ± 1.5 mm^3^ T_4_ 5 μg/kg, 4.0 ± 1.3 mm^3^ T_4_ 50 μg/kg; mean ± SEM) as shown in Fig. 2b. All treatments had no influence on the behavior of sham-operated mice (data not shown).

The doses used in the present studies have been determined in preliminary studies (data not shown). No adverse effects related to hyperthyroidism were seen following any of the given doses. In addition, no differences were observed in body weight or temperature in animals from all groups throughout the studies (Additional file 1: Table S1). In all experimental groups, plasma levels of T_3_ and T_4_ were in physiological range at the endpoint of the study (Additional file 1: Figure S2).

We performed the open field test to ascertain that TH administration was not associated with anxiety or depression-like behavior. Treatment with TH did not affect open field scores, indicative that the treatment did not induce anxiety (Additional file 1: Figure S3).

### Treatment with T_3_ did not affect the expression of TH receptors after PT

To characterize if functional improvement after T_3_ administration was mediated by its binding to respective TR, we assessed their expression in the post-ischemic brain. We found that both isoforms, TRα1 and TRβ1, were ubiquitously expressed in the brain. TR were expressed in the cytoplasm of NeuN and PV^+^ neurons in the peri-infarct region and in GFAP positive reactive astrocytes in the glial scar surrounding the infarct (Fig. 2c). In contrast, CD68 positive monocytic phagocytes and GST-pi positive oligodendrocytes were not immunoreactive for TR (Additional file 1: Figure S4).

Importantly, treatment with T_3_ or T_4_ at 5 or 50 μg/kg did not change the levels of TRα1 (Fig. 2d), despite there was a nonsignificant elevation of TRα1 protein levels found in protein extracts obtained from the peri-infarct area (0.67 ± 0.22 Vh, 0.15 ± 0.05 T_3_, 1.15 ± 0.12 T_4_; arbitrary units, mean ± SEM). Likewise, no changes have been found in TRβ1 levels (1.59 ± 0.51 Vh, 1.61 ± 0.20 T_3_, 1.74 ± 0.42 T_4_; arbitrary units, mean ± SEM) (Fig. 2e).

### Thyroid hormone receptor pattern expression in human stroke patients

Both receptor isoforms were also found in *post-mortem* brain tissues. The levels for both isoforms did not differ between the peri-infarct area from stroke patients and cortex samples from non-stroke patients. However, differences were observed in the infarct core. Here, TRβ1 protein levels increased (0.37 ± 0.02 Ctrl, 0.32 ± 0.03 PI, 0.63 ± 0.07 IC; arbitrary units, mean ± SEM) while levels of TRα1 decreased (0.85 ± 0.13 Ctrl, 0.91 ± 0.16 PI, 0.38 ± 0.15 IC; arbitrary units, mean ± SEM) (Fig. 2f, g).

### Treatment with T_3_ increases dendritic spine density in principal neurons and modulates synaptic neurotransmission

**Fig. 3:**
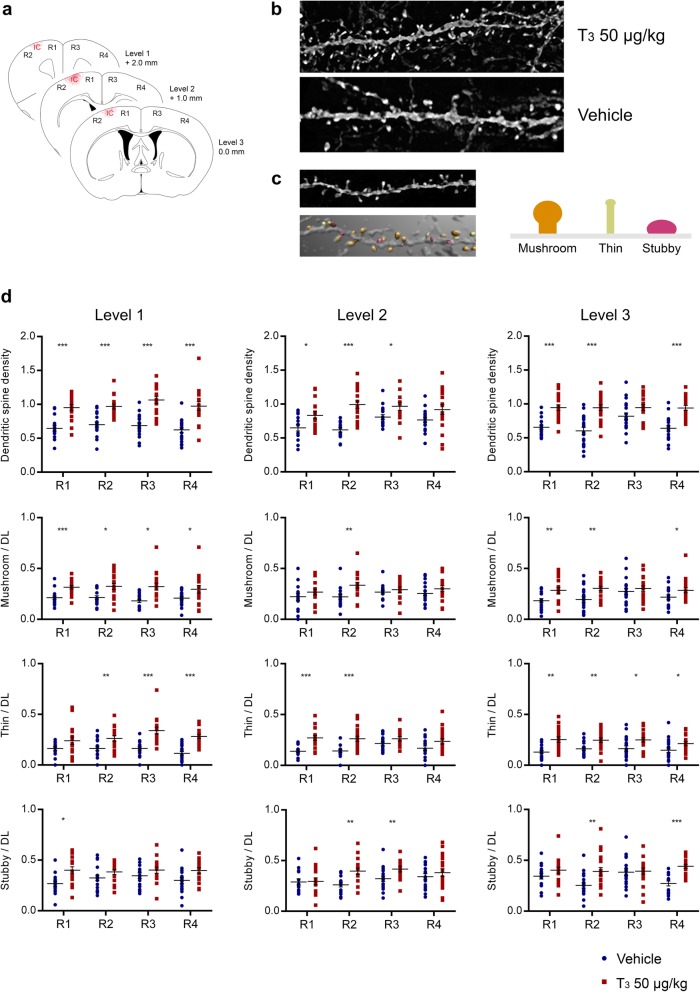
Treatment with T_3_ 50 μg/kg increases dendritic spine density 14 days after photothrombosis (PT). **a** Dendritic spine analysis 14 days after PT at different distances from bregma correspondent to the rostral pole (level 1), center (level 2) and caudal pole (level 3) of the cortical infarct. The regions analyzed correspond to the ipsilateral (R1) and contralateral (R3) motor cortex; and ipsilateral (R2) and contralateral (R4) somatosensory cortex. **b** Representative dendritic segments from animals treated with T_3_ 50 μg/kg (*n* = 4) and Vehicle (Vh; *n* = 4). **c** Apical dendritic spines from cortex layers II/III were automatically detected by NeuronStudio software and classified as mushroom, thin or stubby. Three to five dendritic segments were analyzed per animal. **d** Dendritic spine density (number of total spines / dendritic length) per region and classification of dendritic spines as mushroom, thin or stubby and their density per region, at each level analyzed. Results are displayed as means ± SEM. Statistical analysis was performed with two-tailed unpaired Student *t*-test, *p < 0.05, **p < 0.01, ***p < 0.001

Using Thy1-YFP transgenic mice, we performed a second study to evaluate if T_3_ at 50 μg/kg was involved in modulation of dendritic spine density and morphology as an estimate of structural plasticity in the postischemic brain. The study design including surgeries and treatment with T_3_ at 50 μg/kg or Vh were adopted from *Study I*. To determine the possibility of formation of new synaptic connections 14 days after T_3_ administration, we evaluated dendritic spine density and morphologic classification in four regions corresponding to the peri-infarct area and remote areas to stroke (Fig. 3a).

Infarct volumes did not differ between the treatments (1.0 ± 0.45 mm^3^ Vh, 1.32 ± 0.41 mm^3^ T_3_; mean ± SEM) and did not affect dendritic spines in regions of interest. Representative dendritic branches from mice treated either with T_3_ at 50 μg/kg or Vh are shown in Fig. 3b. Each dendritic spine was classified as mushroom, thin or stubby using the NeuronStudio software (Fig. 3c).

Throughout all three levels covering the anterior, middle and posterior peri-infarct area and homotypic regions of the contralateral hemisphere, the overall number of dendritic spines was increased in T_3_-treated animals compared to Vh-treated animals. In particular, a significant increase in mushroom type spines was observed in R1, level 1, thin spines in R3 and R4 from level 1 and R1 and R2 from level 2 and stubby spines in R2 and R4 of levels 2 and 3 (*p* < 0.001, all regions). Together, we found an increment of dendritic spine density in T_3_-treated animals, in all regions and sections analyzed, particularly in the region correspondent of ipsilateral somatosensory cortex (Fig. 3d).

**Fig. 4:**
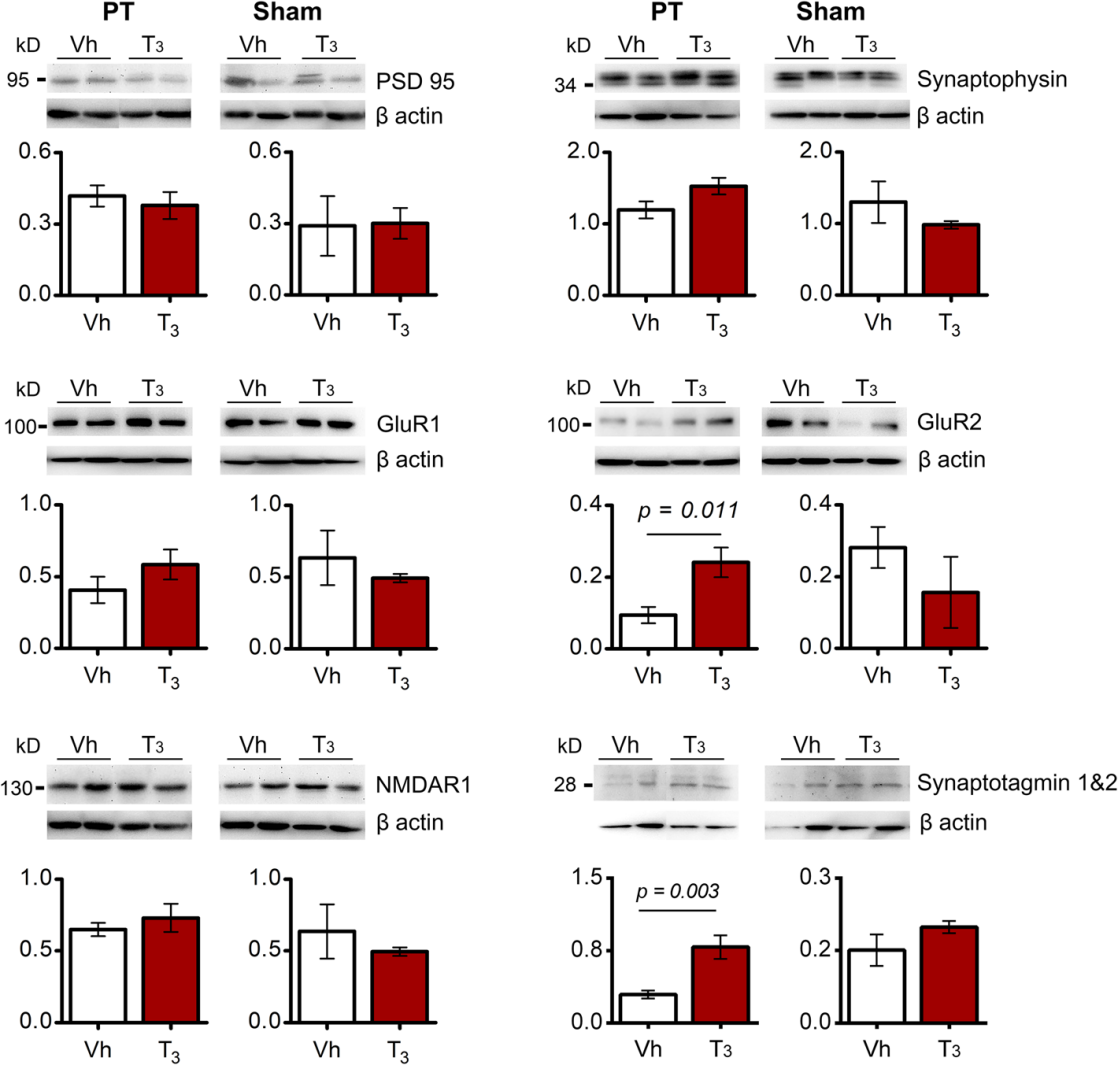
Levels of synaptic proteins in the infarct core and peri-infarct area 14 days after photothrombosis (PT) or in the homotypic area in sham operated mice have been analyzed after treatment with Vehicle (Vh; *n* = 6 for PT and *n* = 3 for sham) or T_3_ 50 μg/kg (*n* = 6 for PT and *n* = 3 for sham). There are no significant differences between levels of postsynaptic density protein 95 (PSD95), synaptophysin, glutamate receptor 1 (GluR1) and NMDA receptor 1 in the infarct core and peri-infarct in T_3_-treated mice compared with Vh. Levels of AMPA receptor subunit GluR2 and synaptotagmin 1&2 are increased in the infarct core and peri-infarct in T_3_-treated mice compared with Vh. Synaptotagmins are vesicle-associated synaptic proteins involved in neurotransmitter release. For uncropped images of western blots see Additional file 1: Figure S6. No differences were observed in sham operated mice. Results are displayed as means ± SEM. Statistical analysis was performed with two-tailed unpaired Student’s *t* test

These findings prompted us to investigate if treatment with T_3_ at 50 μg/kg modulates pre- and/or postsynaptic proteins, which reflects structural changes in dendritic spines and the number of functional synapses relevant for synaptic neurotransmission in the peri-infarct area. We observed no differences in the level of the presynaptic synaptophysin and the PSD95 (Fig. 4). Likewise, no differences were detected in NMDAR1. Interestingly, we found that glutamate receptor 2 (GluR2), one of the AMPA receptor subunits, was significantly increased in mice subjected to PT and treated with T_3_ while levels of GluR1 remained stable (Fig. 4). Accompanied we found increased levels of synaptotagmin 1&2. In sham operated control experiments, treatment with T_3_ had no effect on all studied proteins.

### Synaptotagmins are downregulated by T_3_ in an in vitro model of ischemia and are downregulated in the infarct core of human stroke

**Fig. 5:**
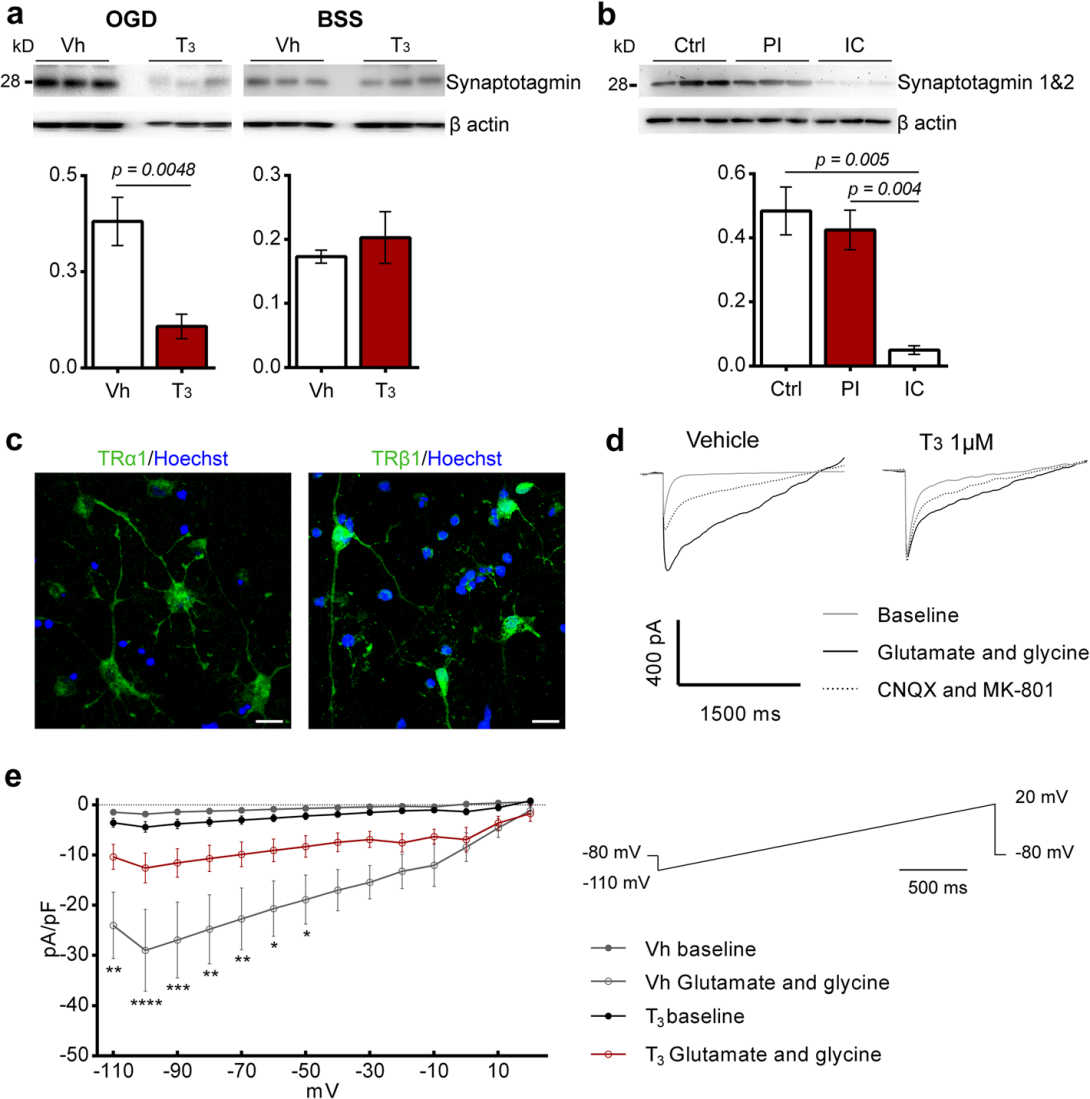
Treatment with T_3_ 1 μM for 48 h inhibits iGluRs evoked currents in cultured cortical glutamatergic neurons and downregulates synaptotagmin levels after oxygen and glucose deprivation (OGD). **a** In an in vitro model of acute cerebral ischemia, levels of synaptotagmin are decreased in cells pre-treated with T_3_ 1 μM for 48 h but no difference was observed in the control conditions (Vh, *n* = 5; T_3_ 1 μM, *n* = 5). **b** Human brains of stroke and non-stroke control cases have been analyzed for levels of synaptotagmin 1&2. Levels of synaptotagmin are decreased in the infarct core (IC) in comparison with control (Ctrl) and peri-infarct (PI) regions (*n* = 3 for each brain region). For uncropped images of western blots see Additional file 1: Figure S7 **c** Representative images of expression of Thyroid hormone receptors TRα1 and TRβ1 (AF488, green) in cultured cortical glutamatergic neurons. TRα1 was mainly localized in the cytoplasm and TRβ1 was expressed in the cytoplasm and nucleus. Scale bar 20 μm. **d** Representative traces obtained during voltage ramps from − 110 to + 20 mV after application of glutamate 50 μM and glycine 3 μM, held at − 80 mV. After application of AMPA and NMDA antagonists, CNQX and MK-801 respectively, currents were almost fully reverted. **e** I-V relationship of glutamate 50 μM and glycine 3 μM induced current in cortical neurons under voltage clamp condition under the membrane potential of − 80 mV. Each trace is the result of the average of three ramps for each 10 mV (Vh, *n* = 3; T_3_ 1 μM, *n* = 4). Results are displayed as means ± SEM. Statistical analysis was performed with two-tailed unpaired Student’s *t* test to compare glutamate induced currents in cells pre-treated with T_3_ 1 μM for 48 h, *p < 0.05, ** *p* < 0.01, *** *p* < 0.001

The finding that T_3_ at 50 μg/kg modulates levels of synaptotagmin 1&2 in vivo prompted us to evaluate its expression in OGD-treated neuronal cultures pre-treated with T_3_ 1 μM for 48 h. Levels of synaptotagmin were significantly decreased in neuronal cultures in the presence of T_3_ (Fig. 5a). This pre-synaptic protein was also expressed in the ischemic territory of stroke patients, being significantly reduced in the infarct core (Fig. 5b).

### T_3_ inhibits glutamate evoked currents in glutamatergic cortical neurons

To study the relevance of T_3_ for neuron function we used the method of voltage ramp to establish information about the I-V relations of calcium permeable NMDA and AMPA post-synaptic receptors in the presence and absence of T_3_. For each cell tested, membrane current amplitudes were normalized in order to obtain current density (pA/pF).

Glutamatergic neurons responsiveness to T_3_ stimulation was consistent with the positive immunoreactivity for TRα1 and TRβ1 (Fig. 5c). Application of agonist glutamate at 50 μM and NMDA co-agonist glycine at 3 μM elicited an inward component at negative potentials. Glycine together with glutamate potentiated the glutamate induced current, even in the presence of Mg^2+^ in the extracellular bath. We also examined the possibility of glycine to induce currents by itself. Application of glycine at 3 μM did not induce a current in any of the neurons tested (Additional file 1: Figure S8). We also tested if response was mediated by postsynaptic iGluRs NMDA and AMPA, by application of non-competitive antagonists MK-801 and CNQX at 10 μM, respectively. After application, currents were almost reversed (Fig. 5d). Similarly, to the application of the antagonists, currents are also almost reversed after washout with extracellular bath (data not shown).

Compared with cells in control conditions, the presence of T_3_ (1 μM) in cell cultures for 48 h before the experiments significantly decreased glutamate / glycine response in the neurons analyzed (Fig. 5e).

### T_3_ downregulates GABA synthesis and activity of cortical Parvalbumin immunoreactive cells

**Fig. 6:**
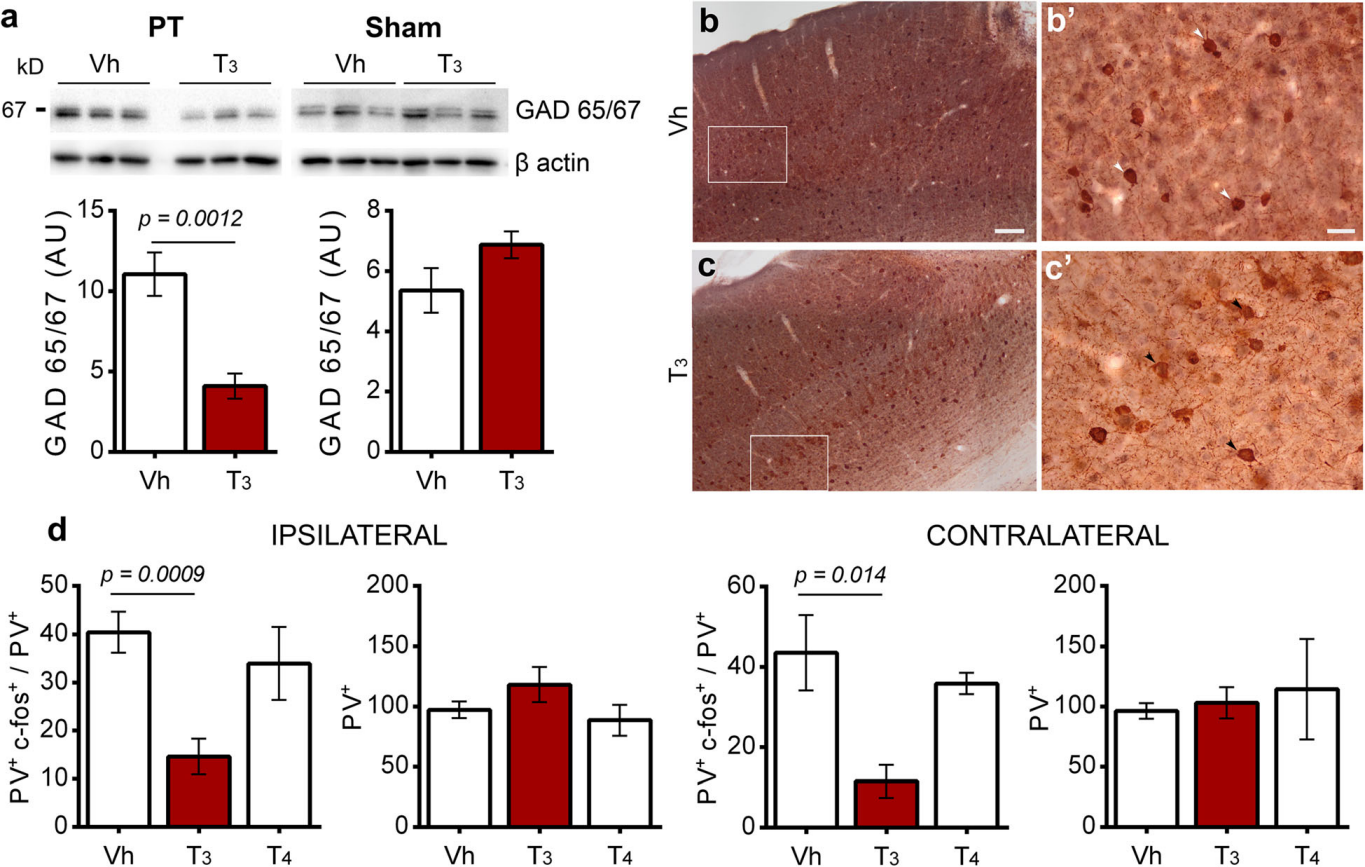
Treatment with T_3_ 50 μg/kg is associated with downregulation of GABA synthesis in the infarct core and peri-infarct area 14 days after photothrombosis (PT) and the activity of cortical parvalbumin immunoreactive cells (PV^+^) in the ipsilateral and contralateral areas. **a** and **b** Immunohistochemistry NiDAB (c-fos) counterstained with NovaRed (PV) was performed to count PV^+^ c-fos^+^ (white arrows) and PV^+^ (black arrows) immunoreactive cells, in the ipsilateral and contralateral motor and somatosensory areas of mice treated with Vehicle (Vh) or T_3_ 50 μg/kg. Scale bars 100 μm (**a** and **b**) and 20 μm (**a’** and **b’**). **c** Levels of glutamate decarboxylase (GAD) 65/67 was downregulated in mice treated with T_3_ 50 μg/kg, in the peri-infarct area 14 days after PT. Vh (*n* = 6 for PT and *n* = 3 for sham), T_3_ 50 μg/kg (*n* = 6 for PT and *n* = 3 for sham). For uncropped images of western blots see Additional file 1: Figure S9. Results are displayed as means ± SEM. Statistical analysis was performed with two-tailed unpaired Student’s *t* test. **d** Functional recovery after T_3_ 50 μg/kg treatment may be related to a decrease in the ratio between PV^+^ c-fos^+^ / PV^+^ immunoreactive cells observed in the ipsilateral area and the correspondent region in the contralateral hemisphere. Vh (*n* = 7), T_3_ 50 μg/kg (*n* = 6), T_4_ 50 μg/kg (*n* = 3). Results are displayed as means ± SEM. Statistical analysis was performed by One-way ANOVA and Bonferroni’s multiple comparisons test. Two-tailed unpaired Student’s *t* test was employed to determine *p* values

To determine whether functional recovery mediated by i.p. injection of T_3_ at 50 μg/kg modulates GABAergic signaling, we evaluated GAD 65/67 expression in stroke mice treated with T_3_ compared with Vh. Longterm administration of T_3_ 50 μg/kg for 14 days after ischemic stroke significantly reduced GAD 65/67 expression in the ischemic territory. In sham operated animals, administration of T_3_ did not alter the expression of GAD 65/67 (Fig. 6a).

To understand the significance of lower GAD 65/67 expression in animals treated with T_3_, we assessed the activity of cortical PV neurons, a class of interneurons that regulate GABA neurotransmission. PV immunoreactivity was co-localized with the activity-dependent marker c-fos, through NovaRED Peroxidase (PV) and DAB (c-fos) immunohistochemistry (Fig. 6b, c). As shown in Fig. 6d, there is a significant reduction in PV^+^ / c-fos^+^ ratio between Vh and T_3_ 50 μg/kg-treated animals in the peri-infarct region (40.46 ± 4.26 Vh; 14.62 ± 3.4 T_3_ 50 μg/kg; mean ± SEM) and the homotypic region in the contralateral hemisphere (43.61 ± 9.43 Vh, 11.54 ± 4.12 T_3_ 50 μg/kg; mean ± SEM). In contrast, treatment with T_4_ 50 μg/kg did not change the activity of PV^+^ cells in the same regions (Ipsilateral 33.97 ± 7.59; Contralateral 35.91 ± 2.65; mean ± SEM). Importantly, treatment with TH did not influence the total number of PV immunoreactive cells in the ipsilateral and contralateral hemispheres (Fig. 6d).

## Discussion

After an ischemic stroke, there is a disruption of normal neuron function i.e. synaptic activity due to cell death occurring in the infarct core and therefore, disruption in the normal neuronal circuity [87]. As consequence, surviving neurons adjacent to the infarct spontaneously adopt homeostatic mechanisms that contribute to maintain overall excitability, although to a limited extent [18, 21, 48]. The molecular mechanisms of homeostatic processes characterize the recovery phase of ischemic stroke and enhancing those with adjuvant interventions might be a key therapeutic strategy [19]. This may create a wider therapeutic window to optimize and restore lost neurological function.

TH have been recently proposed as a key modulator in stroke [71] and brain injury recovery [41]. The lacking evidence of the underlying mechanisms of TH promoting functional recovery after stroke prompted us to evaluate the role of TH in the post-ischemic brain. Summarizing, our work demonstrates for the first time that T_3_ modulates key homeostatic regulatory mechanisms that are crucial to maintain appropriate levels of excitation and mechanisms that stabilize neuronal activity in the post-ischemic brain, contributing to cortical reorganization and to functional recovery.

Given that TH signaling could be related to better outcome, we first assessed behavioral recovery after experimental stroke in mice treated with T_4_ or T_3_ at 5 or 50 μg/kg. The photothrombotic model adopted for our study induced a well-defined ischemic damage in the primary motor cortex that produced consistent hemiparesis 2 days after stroke [53], allowing behavioral assessment of motor function following ischemia. As expected, all mice spontaneously recovered some of lost motor function over time in analogy to spontaneous recovery in humans [23, 24]. Interestingly, the group treated with T_3_ 50 μg/kg had significant higher neurological scores 14 days after PT, with no difference in the infarct size compared to control group. However no significant statistical differences were observed in the T_4_-treated mice groups. T_4_ is the prohormone and it needs to be converted to T_3_ before it can exert any biological effect [47]. In the rodent, half of T_3_ levels in the brain is provided from its free fraction in blood circulation and cerebrospinal fluid and the other half relies in local deiodination of T_4_ in astrocytes and tanycytes, which concentration is regulated by deiodinases activity [44, 74]. Although we did not verify deiodinase expression in the post ischemic brain, the possible scenario is that administration of T_4_ is less effective to exert action in the brain, since it still needs to be converted to the active form T_3_.

Next, we investigated the key T_3_-mechanisms that might contribute for stroke recovery. Taking into account that genomic actions of T_3_ in the brain are mainly mediated by binding to TRα1 and TRβ1 [71], we assessed their levels and expression pattern in the post-ischemic mouse brain. TR levels were not altered after administration of T_4_ or T_3_ at 50 μg/kg, suggesting that recovery induced by T_3_ was mediated by other mechanisms. However, our results do not exclude the possibility that genomic actions in the brain have an impact on stroke recovery also at different temporal and spatial scales, in other animal models or in humans. Indeed, one study reported a reduction of TRβ1 expression in the infarct core compared with unaffected peri-infarct cortex and contralateral hemisphere 14 days after permanent middle cerebral occlusion (MCAO) [43]. We also found that TRβ1 was significantly increased in the infarct core in the human brain, when compared to non-stroke patients. Taken together, we show that cerebral ischemia induces heterogenic changes in human brain TR expression, which may imply an important role for T_3_ signaling.

Although TR are mainly nuclear, TRα1 and TRβ1 have been also found in the cytoplasm, which may increase T_3_ nuclear import [2]. Interestingly, we observed that TRα1 and TRβ1 was heterogeneously expressed in the cytoplasm of neurons and in reactive astrocytes from the glial scar, in accordance with a previous study performed 14 days after MCAO [43]. However, none of TR isoforms were found in positive GFAP astrocytes in the naïve rodent brain [14]. If TR expression has implications in the formation and function of the glial scar should be the subject for subsequent studies.

Besides genomic actions, other TH-mediated non-genomic mechanisms may contribute for stroke [71] and brain injury [41] recovery. After ischemic stroke, there is an extensive and rapid loss of neurons and degeneration of their axons and dendritic spines in remote areas [87], in both ipsilateral and contralateral cortex [31], leading to a disruption in normal function of neuronal circuits and loss of brain function. In analogy to brain development and learning/plasticity mechanisms, surviving neurons after stroke attempt to stabilize the ratio between excitatory – inhibitory circuits, in order to adjust brain excitability [21]. A wide variety of homeostatic mechanisms might contribute to the maintenance of overall excitability, involving the regulation of neuronal intrinsic excitability and synaptic transmission [52, 76, 77]. Here, we have identified for the first time T_3_-modulated mechanisms of homeostatic plasticity that were related to motor recovery after experimental stroke. In particular, we have shown that T_3_ modulates plasticity mechanisms that may operate on different temporal and spatial scales as compensatory mechanisms to assure appropriate synaptic neurotransmission.

Dendritic spines are highly dynamic [6, 84] and especially after stroke it occurs an extensive reorganization in dendritic arbors, which includes an increase in spine density and spine turnover [12, 13, 25], particularly in apical cortical pyramidal neurons within the first 2 weeks [11]. In *Study II* we observed overall enhanced cortical reorganization in T_3_-treated Thy1-YFP mice reflected in increased spine density in cortical layers II/III, especially in the peri-infarct area, which may contribute for spontaneous recovery. The process of spine formation or spinogenesis includes the formation of thin and long dendritic filopodia that are highly dynamic and establish contact with presynaptic axons. The presence of appropriate signals would result in stabilization of the contact and maturation of filopodia into functional dendritic spines [6]. Interestingly, we found increased density of thin protrusions in T_3_-treated animals, especially in the peri-infarct area, although in a temporal scale we could not distinguish newly formed protrusions from the pre-existing ones. We also observed an increased number of mushroom-like spines in the peri-infarct region in all sections analyzed from T_3_-treated mice. Although we could not assure that all protrusions are or will be transformed in more stable thin or mushroom-like spines over time, this was a direct finding that T_3_ modulated the reorganization of spines in numbers and structure 2 weeks after stroke onset.

Based on these findings, we further evaluated synaptic efficacy. To address this question, we studied levels of pre-synaptic proteins synaptophysin and synaptotagmin, important to regulate endocytosis and exocytosis of synaptic vesicles, respectively [39, 68, 69] and therefore neurotransmitter release. In particular, synaptotagmins are crucial for the docking of synaptic vesicles and fusion with neuron membrane [69]. We demonstrate that in the human ischemic infarct core, levels of synaptotagmin 1&2 were very low due to cell death and loss of synaptic neurotransmission. Nevertheless, their levels in the peri-infarct remained as the same as non-stroke brain tissue, which makes synaptotagmin a molecular target. The increase in synaptotagmin 1&2 levels in the post-ischemic brain of T_3_-treated mice supports an increase of neurotransmitter release probability, which in turn may increase synaptic efficacy [9]. In contrast, we observed that synaptotagmin is reduced in OGD T_3_-treated cultured glutamatergic neurons, which demonstrated homeostatic regulation by T_3_ in order to reduce neurotransmitter release and hyperexcitability in an in vitro model of acute brain ischemia. Synaptotagmin related gene 1 is a TH responsive gene during brain development, regulating synaptic activity and structure [73] and T_4_ has been reported to restore synaptotagmin 1 levels to normal in hypothyroid rats [83]. However, how T_3_ activates / inhibits synaptic vesicles for synaptotagmin action remains to be elucidated.

Besides neurotransmitter release, efficacy of neurotransmission is dependent on post-synaptic response to glutamate in neuron terminals, that can be modulated by changing the number or function of iGluRs AMPA and NMDA [10, 51, 75, 76]. Indeed, stroke-induced glutamate release activates AMPA receptors [17] and NMDA receptors [54], changes that are related with excitatory synaptic transmission and motor recovery. Here we show an increase in levels of AMPA receptor subunit GluR2 in the peri-infarct area of mice treated with T_3_. The AMPA receptor subunit GluR2 regulates critical aspects of AMPA receptor function, neurotransmission and synaptic plasticity [32, 66] which ultimately contributes to increased excitability in the post-ischemic brain and recovery [67].

We characterized AMPA and NMDA excitatory post-synaptic currents with a voltage-clamp method in cultured glutamatergic neurons pre-treated for 48 h with T_3_ 1 μM. Interestingly, we found that glutamate evoked currents were significantly lower in neurons previously incubated with T_3_. Similarly, in a previous study, T_3_ at 10 μM has been implicated in the reduction of miniature excitatory post-synaptic currents frequency and glutamate induced toxicity in hippocampal neurons [42]. Interestingly, we found that T_3_ recruits divergent mechanisms to achieve homeostasis in two different systems regarding synaptic network organization, i.e., in vitro and in vivo and dependent on the activation status of neurons and brain tissues, respectively. Important for stroke recovery, T_3_ could modulate synaptic neurotransmission to an optimal firing rate.

After an ischemic insult, synaptic glutamate signaling is depressed also due to tonic inhibition of neuronal circuits, which ultimately restricts the process of recovery [8, 15, 80]. Modulation to shift the excitation - inhibition ratio by stimulation of glutamate signaling [15, 17] and reducing GABA inhibition [1, 15, 16] in the motor and somatosensory cortex accelerates motor recovery in mice. GABAergic neurotransmission is mediated by cortical interneurons, a group of cells expressing calcium-binding proteins, including PV. In fact, a correlation between reduction of PV/GABA cells and functional recovery in rodents subjected to stroke has been shown [86]. Also, different therapeutic approaches such as environment enrichment [29], benzodiazepine inverse agonist [1], but also intravenous infusion of human bone marrow mesenchymal stromal cells after transient MCAO [62] decreased cortical PV immunoreactivity or activity and were associated with enhanced recovery of lost neurological function. Treatment with T_3_ reduced the activity of PV immunoreactive cells in the peri-infarct area and in the contralateral hemisphere, without affecting the total number of PV^+^ cells.

Concomitantly, in the peri-infarct area of animals treated with T_3_ 50 μg/kg, expression levels of GAD 65/67 was significantly reduced, and directly GABA production. Our results are in accordance with studies describing an increased GAD activity and GABA uptake in neurons in hypothyroid state [34] and the finding that T_3_ administration inhibits GABA-induced Cl^−^ currents [45]. Thus, the decrease in PV cortical activity may facilitate experience dependent plasticity and decrease GABA availability and tonic inhibition, and therefore contribute to restoration of neuronal networks.

**Fig. 7:**
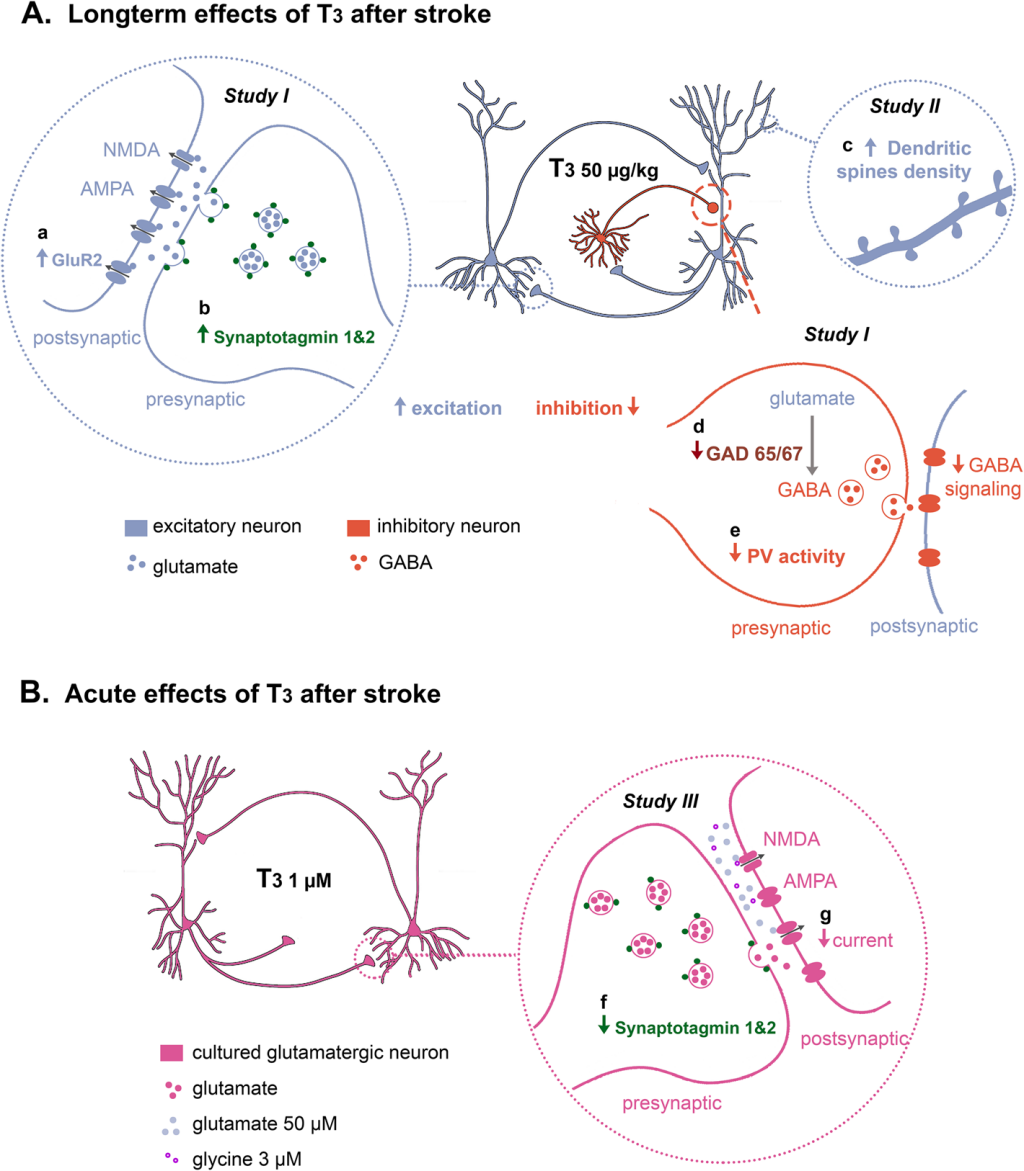
Proposed mechanisms of homeostatic regulation of neurotransmission by T_3_. (A) After photothrombosis (PT), administration of T_3_ 50 μg/kg for 14 days modulates pathways during the recovery period after stroke involved in reorganization of neuronal circuits and synaptic plasticity, to balance excitation and inhibition ratio. **a** T_3_ increases levels of post-synaptic glutamate receptor 2 (GluR2) subunit in AMPA receptors in the peri-infarct area and **b** increases levels of synaptotagmin 1&2, increasing the probability of neurotransmitter release. **c** T_3_ increases dendritic spines density in the ipsilateral and contralateral regions. **d** T_3_ decreases tonic GABAergic signaling in the peri-infarct area by a decrease in the levels of GAD 65/67 and **e** reduced parvalbumin (PV) activity. (B) In an acute model of cerebral ischemia and hyperexcitability, **f** in glutamatergic neurons pre-treated with T_3_ at 1 μM for 48 h there is a decrease in levels of synaptotagmin and **g** T_3_ modulates neuron membrane properties with the balance of inward glutamate ligand-gated channels currents

Together, our findings reveal important implications of T_3_-mediated mechanisms in stroke recovery (Fig. 7). At the cellular and structural level, we demonstrated that T_3_ is involved in mechanisms of neuronal plasticity that collectively contributed to functional recovery following experimental stroke. Based on our findings it will be possible to develop specific approaches targeting T_3_-mediated mechanisms in the post-ischemic brain. Those may result in specific treatments to be tested in clinical trials.

## Supplementary information

**Additional file 1.** Supplementary Methods and Results.
