# Supplementary material for: Triiodothyronine modulates neuronal plasticity mechanisms to enhance functional outcome after stroke
Source: Acta Neuropathol Commun. 2019 Dec 21;7:216. doi: 10.1186/s40478-019-0866-4 (PMC6925884; doi:10.1186/s40478-019-0866-4)
Supplement: Supplementary file 5 — Additional file 1. Supplementary Methods and Results. [file 40478_2019_866_MOESM1_ESM.pdf]

Online Resource 1 Supplemental Material

Triiodothyronine modulates neuronal plasticity mechanisms to enhance functional outcome after stroke

Daniela Talhada[1,2], Joana Feiteiro[2], Ana Raquel Costa[2], Tiago Talhada[1], Elisa Cairrão[2], Tadeusz Wieloch[1], Elisabet Englund[3], Cecília Reis Santos[2], Isabel Gonçalves[2], Karsten Ruscher[1,4*]

[1]Laboratory for Experimental Brain Research, Division of Neurosurgery, Department of Clinical Sciences, Lund University, Sweden.

[2]CICS-UBI-Health Sciences Research Centre, Faculdade de Ciências da Saúde, Universidade da Beira Interior, Covilhã, Portugal.

[3]Division of Oncology and Pathology, Lund University Hospital, Lund, Sweden.

[4]LUBIN Lab - Lunds Laboratorium för Neurokirurgisk Hjärnskadeforskning, Division of Neurosurgery, Department of Clinical Sciences, Lund University, Sweden.

*__Corresponding Author:__ Dr. Karsten Ruscher, Associate Professor, Laboratory for Experimental Brain Research, Wallenberg Neuroscience Center, Lund University, BMC A13, S-22184 Lund, Sweden. Tel +46-46-2220613. Fax +46-46-2220615. email: karsten.ruscher@med.lu.se

This PDF includes:

Supplementary Figures 1-9

Supplementary Table 1

Legends for Supplementary Videos 1-4

Supplementary Methods

Other Supplementary material for this manuscript includes:

Supplementary Videos 1-4

Supplementary Figures

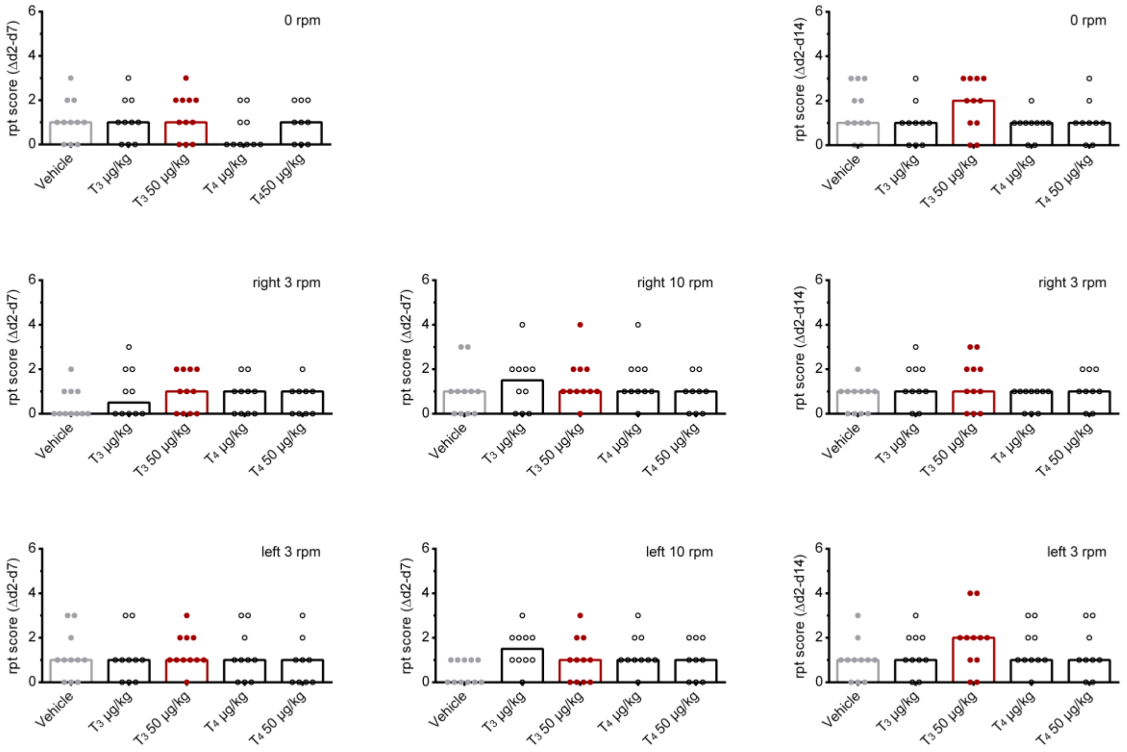

Supplementary Fig. 1

Rotating pole test after photothrombosis (PT). Difference between the rotating pole test (rpt) scores from day two (selective sorting) and seven (Δd2-d7) and from day two and 14 (Δd2-d14) at 0, 3 and 10 rotations per minute (rpm) to the right and to the left sides, from mice subjected to PT (right hemisphere). Scores are shown as individual data and group median. Statistical analysis was performed by Kruskal-Wallis test followed by Mann-Whitney test. Vehicle (*n* = 11), T_3 5 µg/kg (*n* = 10), T_3 50 µg/kg (*n* = 11), T_4 5 µg/kg (*n* = 10), T_4 50 µg/kg (*n* = 9)

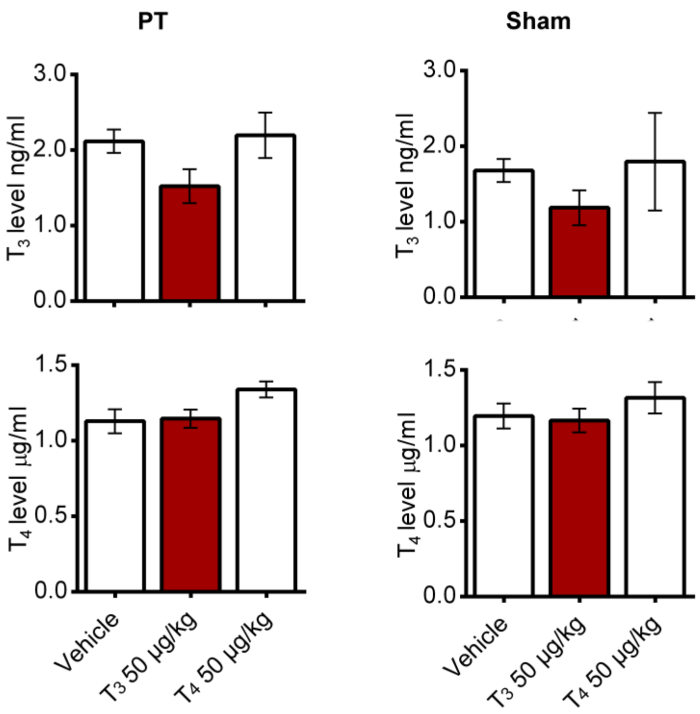

Supplementary Fig. 2

Levels of thyroid hormones in plasma collected from mice 14 days after photothrombosis (PT) or sham surgeries were analyzed after treatment with vehicle, T_3 50 µg/kg or T_4 50 µg/kg (n = 6 for PT and n = 3 for sham, for each condition). Results are displayed as means ± SEM. No statistical differences were seen among experimental groups. Statistical analysis was performed by One-way ANOVA and Bonferroni's multiple comparisons test

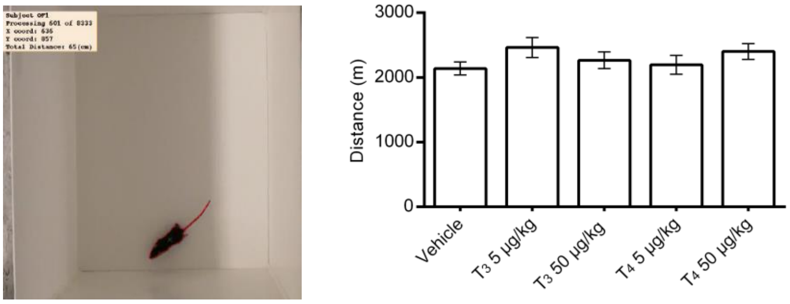

Supplementary Fig. 3

Locomotor activity in the open field test. The total distance traveled by mice did not differ among treatment groups. Statistical analysis was performed by One-way ANOVA and Bonferroni's multiple comparisons test. Data are expressed as mean ± SEM. Vehicle (*n* = 15), T_3 5 µg/kg (*n* = 13), T_3 50 µg/kg (*n* = 13), T_4 5 µg/kg (*n* = 10), T_4 50 µg/kg (*n* = 11)

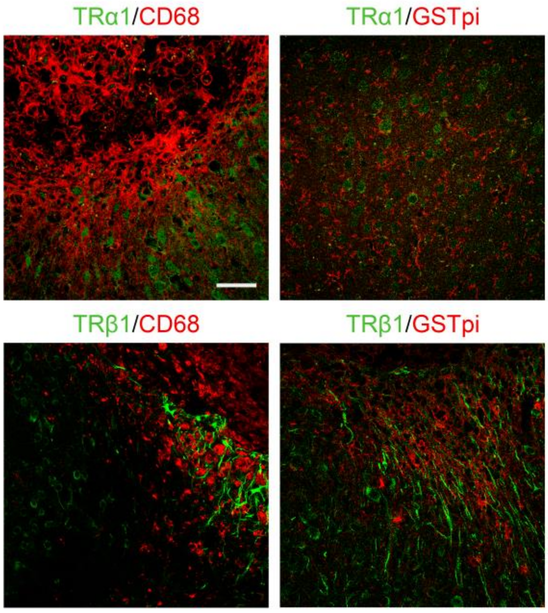

Supplementary Fig. 4

Thyroid hormone receptors (TR) α1 and TRβ1 (AF488, green) expression in mouse brain 14 days after photothrombosis and treated with vehicle (NaCl, 0.9 %). We did not find co-expression in both TR isoforms in CD68 positive microglia (Cy3, red) or GSTpi positive olygodendrocytes (Cy3, red). Scale bar 50 µm

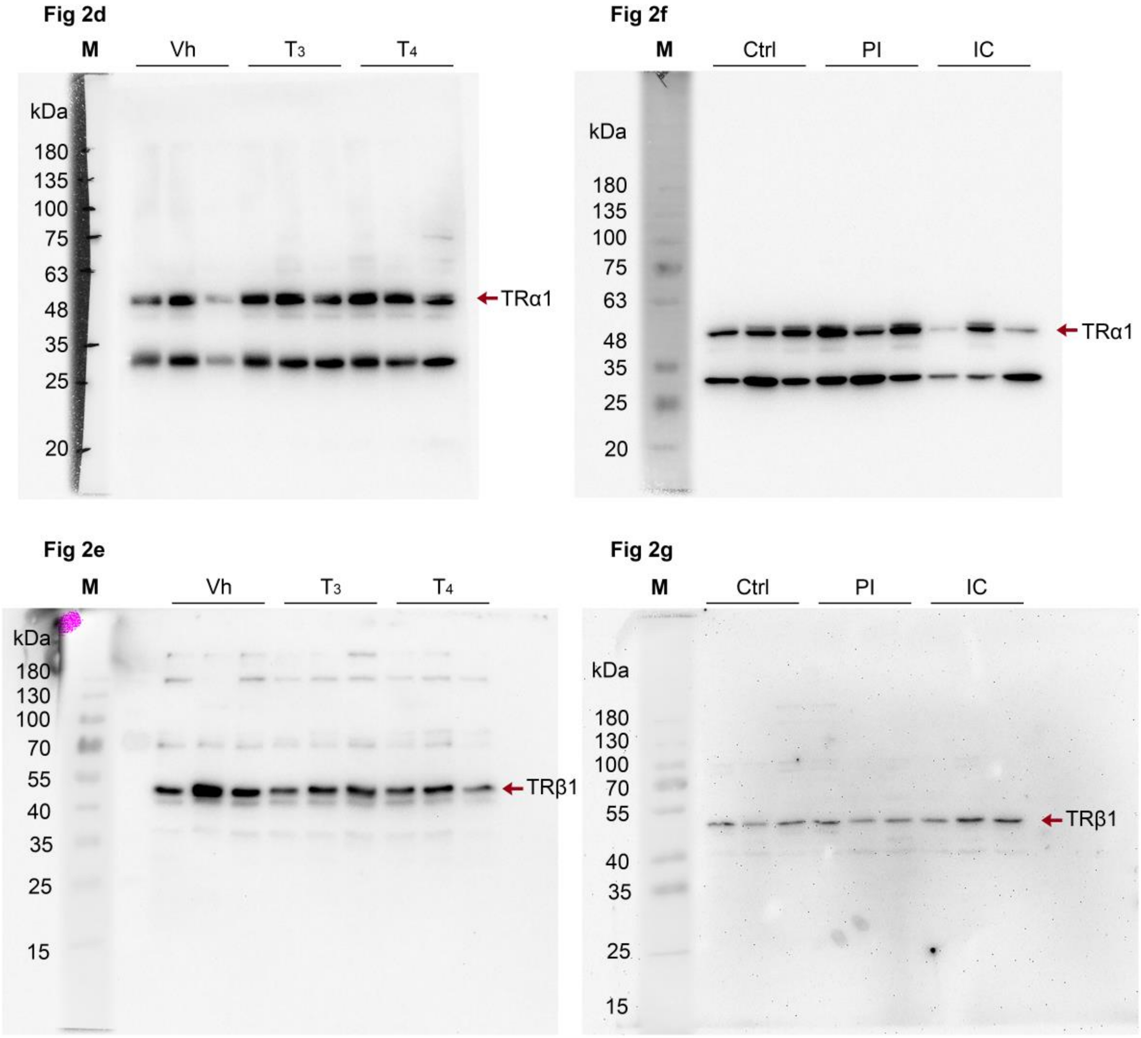

Supplementary Fig. 5

Corresponding uncropped images of western blots shown in Fig. 2 panels d, e, f and g. The protein analyzed is depicted with an arrow

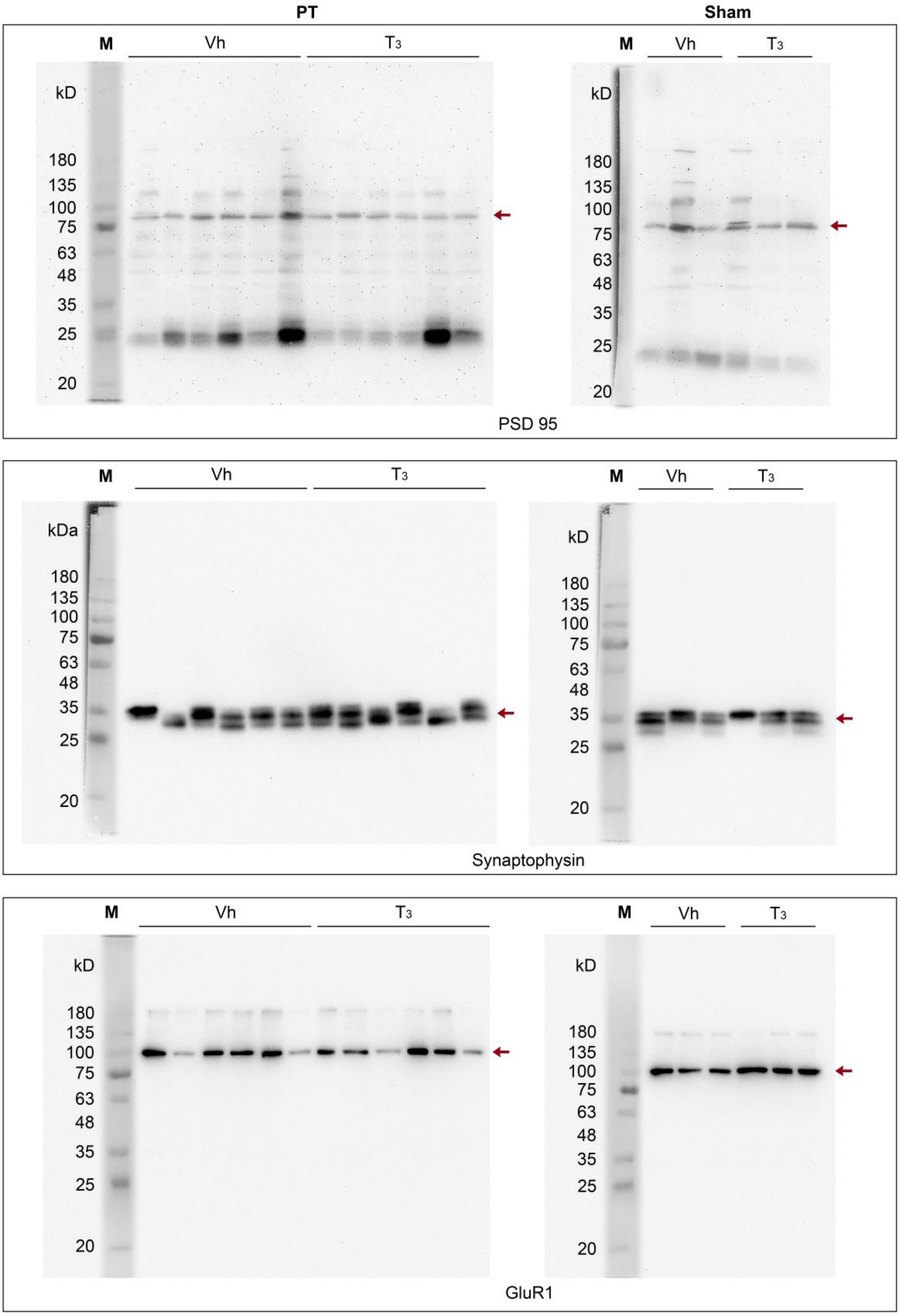

Supplementary Fig. 6 (continue next page)

Corresponding uncropped images of western blots shown in Fig. 4. The protein analyzed is depicted with an arrow

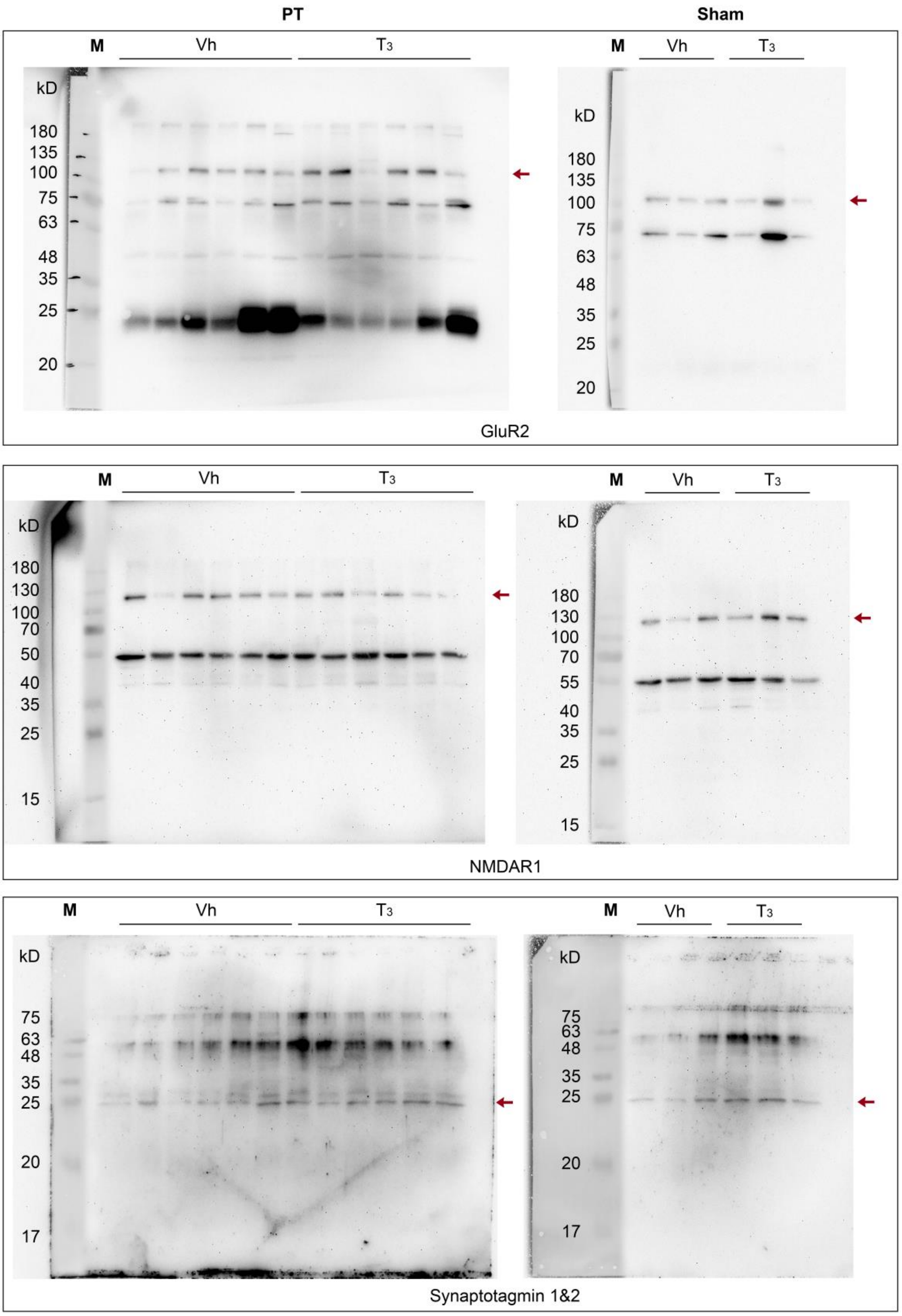

Supplementary Fig. 6

Corresponding uncropped images of western blots shown in Fig. 4. The protein analyzed is depicted with an arrow

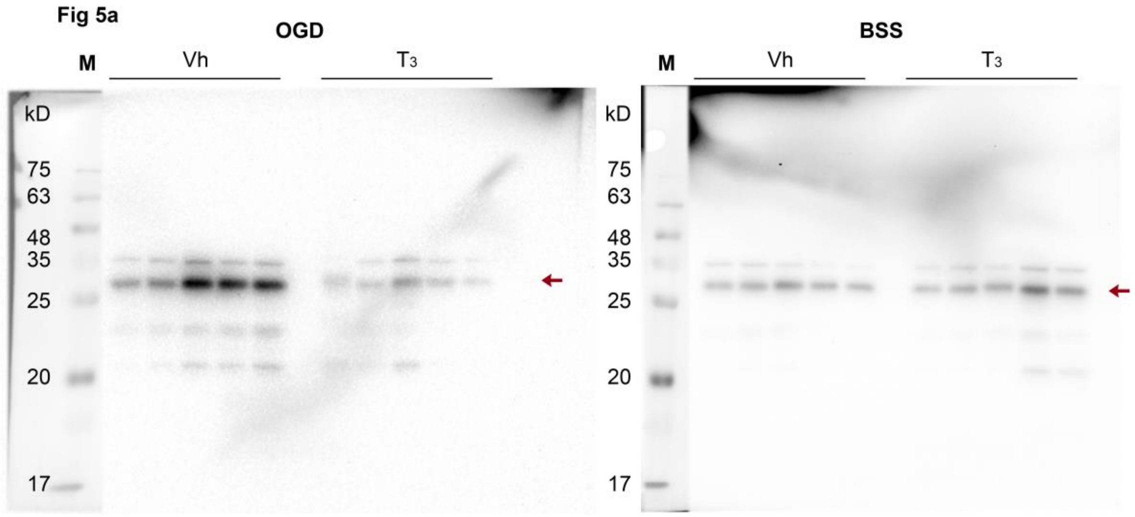

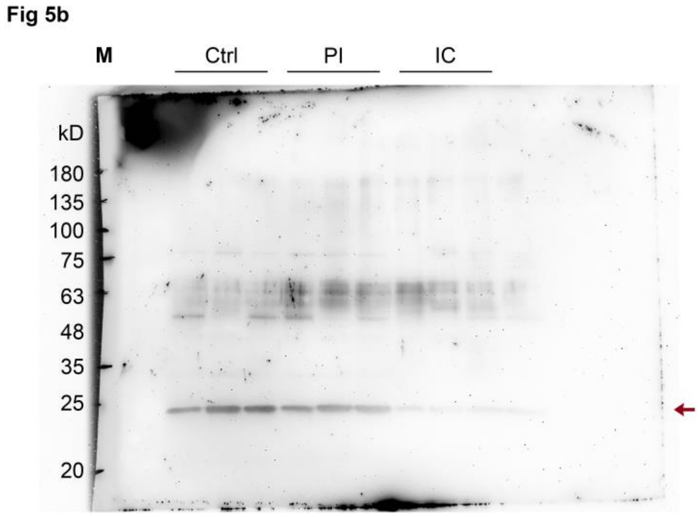

Supplementary Fig. 7

Corresponding uncropped images of western blots for synaptotagmin shown in Fig. 5. The protein analyzed is depicted with an arrow

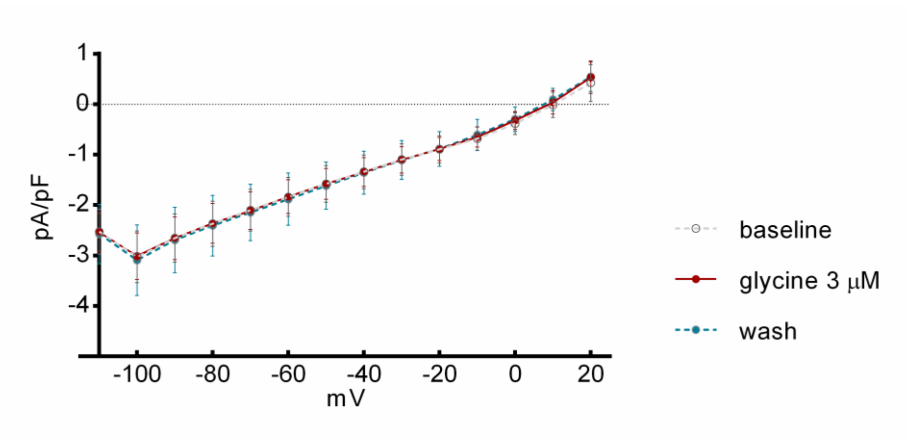

Supplementary Fig. 8

Glycine did not induce inward currents in cultured glutamatergic neurons (*n* = 3). Representative traces obtained during voltage ramps from -110 to + 20 mV after application of glycine 3 μM, held at -80 mV. Results are displayed as means ± SEM

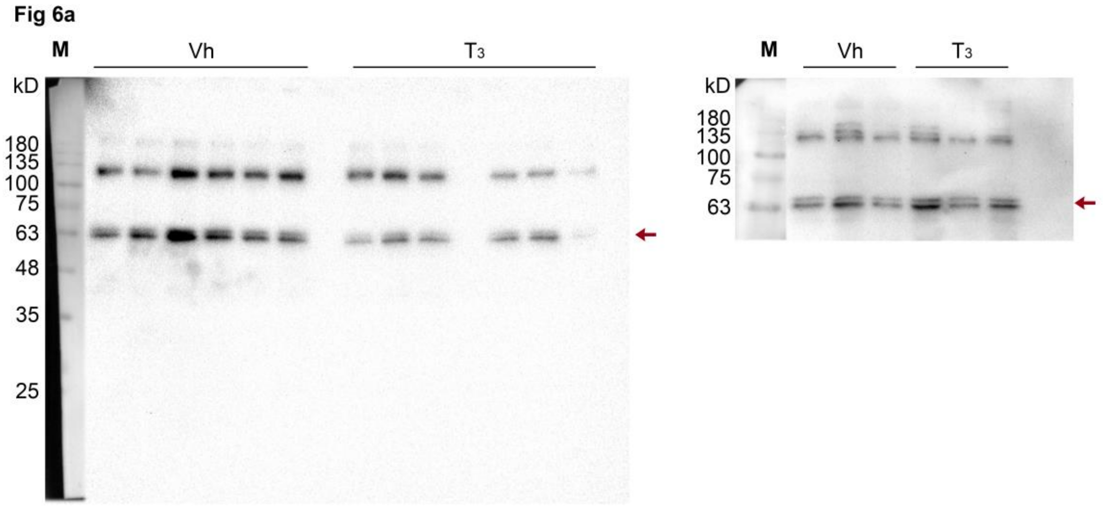

Supplementary Fig. 9

Corresponding uncropped images of western blots shown in Fig. 6. The protein analyzed GAD 65/67 is depicted with an arrow

Supplementary Video legends

Supplementary Video 1. Selective sorting two days after photothrombosis. The mouse could not transverse the pole at 10 rotations per minute to the left, with a final score of 2.

Supplementary Video 2. The same mouse from Video 1 was treated with vehicle (NaCl 0.9) during 14 days after photothrombosis. The mouse could not transverse the pole at 10 rotations per minute to the left, with a final score of 2.

Supplementary Video 3. Selective sorting two days after photothrombosis. The mouse could not transverse the pole at 10 rotations per minute to the left, with a final score of 2.

Supplementary Video 4. The same mouse from Video 3 was treated with T_3 50 µg/kg during 14 days after photothrombosis. The mouse was able to transverse the pole at 10 rotations per minute to the left, with a final score of 5.

Supplementary Table 1. List of body weights (BW) and temperatures (Temp) from animals included in our experimental studies, before and after days 2, 7 and 14 of photothrombosis (PT) or sham surgeries. Results are displayed as means ± SEM

TREATMENT GROUPS			Before		Day 2		Day 7		Day 14	
			BW g (mean ± SEM)	Temp ºC (mean ± SEM)	BW g (mean ± SEM)	Temp ºC (mean ± SEM)	BW g (mean ± SEM)	Temp ºC (mean ± SEM)	BW g (mean ± SEM)	Temp ºC (mean ± SEM)
Study I **PT**	Vehicle	(*n* = 17)	23.9 ± 0.4	37.6 ± 0.1	22.5 ± 0.4	37.6 ± 0.2	23.3 ± 0.4	38.1 ± 0.1	23.7 ± 0.4	37.9 ± 0.2
	T_3 5 µg/kg	(*n* = 14)	23.3 ± 0.5	37.5 ± 0.1	21.7 ± 0.4	37.7 ± 0.2	22.1 ± 0.4	38.3 ± 0.2	22.8 ± 0.4	38.0 ± 0.2
	T_3 50 µg/kg	(*n* = 15)	24.0 ± 0.4	37.4 ± 0.2	22.3 ± 0.4	37.4 ± 0.1	23.4 ± 0.3	38.3 ± 0.1	24.7 ± 0.3	37.8 ± 0.1
	T_4 5 µg/kg	(*n* = 13)	23.9 ± 0.5	37.6 ± 0.1	22.4 ± 0.4	37.5 ± 0.1	23.1 ± 0.4	38.0 ± 0.2	23.5 ± 0.4	38.0 ± 0.1
	T_4 50 µg/kg	(*n* = 16)	23.8 ± 0.3	37.7 ± 0.1	22.5 ± 0.3	37.7 ± 0.1	23.2 ± 0.3	38.2 ± 0.1	23.7 ± 0.3	37.8 ± 0.1
Study I **Sham**	Vehicle	(*n* = 6)	24.1 ± 0.5	37.1 ± 0.3	23.3 ± 0.6	37.5 ± 0.2	23.8 ± 0.6	38.0 ± 0.3	24.3 ± 0.7	38.0 ± 0.4
	T_3 5 µg/kg	(*n* = 6)	24.2 ± 0.9	37.3 ± 0.2	23.1 ± 1.1	37.7 ± 0.1	23.7 ± 1.0	37.7 ± 0.3	24.3 ± 1.1	38.2 ± 0.4
	T_3 50 µg/kg	(*n* = 6)	23.6 ± 0.4	38.0 ± 0.3	23.0 ± 0.6	37.5 ± 0.2	24.0 ± 0.6	38.0 ± 0.1	25.5 ± 0.9	37.9 ± 0.1
	T_4 5 µg/kg	(*n* = 6)	23.4 ± 0.5	37.2 ± 0.3	22.2 ± 0.7	37.3 ± 0.2	22.9 ± 0.6	37.9 ± 0.3	23.3 ± 0.7	37.7 ± 0.3
	T_4 50 µg/kg	(*n* = 6)	23.5 ± 0.3	37.1 ± 0.1	22.5 ± 0.3	37.3 ± 0.2	23.1 ± 0.4	37.9 ± 0.3	23.5 ± 0.5	37.8 ± 0.3
Study II	Vehicle	(*n* = 4)	25.3 ± 0.2	36.9 ± 0.1	23.6 ± 0.3	37.5 ± 0.2	24.6 ± 0.4	38.0 ± 0.2	24.6 ± 0.4	38.0 ± 0.04
	T_3 50 µg/kg	(*n* = 4)	25.7 ± 0.3	37.3 ± 0.2	24.9 ± 0.5	37.7 ± 0.1	25.7 ± 0.6	38.7 ± 0.2	26.2 ± 0.4	38.1 ± 0.3

Supplementary Methods

Mice

Mice were bred and genotyped at conventional facility of Biomedical Centre, (BMC, Lund, Sweden). All animal experiments (*Studies I and II*) were carried in accordance with the international guidelines on experimental animal research and with the approval of the Malmö-Lund Ethical Committee (ethical permit no. M50/2015) and followed the ARRIVE guidelines. Animals were housed in a controlled environment with a 12:12 hour light cycle, room temperature of 22°C and food and water *ad libitum*. In all animal studies, body weight was monitored every day and body temperature was monitored before and on days two, seven and 14 after the surgeries. The studies were performed pre-specified, subjected to randomization and performed in a blinded fashion to the investigator who performed behavior assessments and dendritic spine analysis. All *in vitro* experiments (*Study III*) were carried out in compliance with directives on animal experimentation (Decreto-Lei 113/2013 and 2010/63/EU) in Portugal and European Union and with approval of the comitte of Animal Research at Universidade da Beira Interior (CICS-UBI, Covilhã, Portugal). Human brain tissue used in this study were carried out with the approval of the Lund Ethical Review Board for research involving humans (Dnr 2011/80).

Drugs and drug delivery

Drugs used in this study were T_3 and T_4 (Sigma-Aldrich, Deisenhofen, Germany), glutamic acid (Sigma-Aldrich), glycine (Fisher Scientific), 6-cyano-7-nitroquinoxaline-2,3-dione (CNQX; Sigma-Aldrich) and Dizocilpinehydrogen maleate (MK-801; Sigma-Aldrich). For intraperitoneal administration in mice (*Study I and Study II*), T_3 and T_4 stocks were dissolved in NaOH and diluted in NaCl 0.9% at the desired concentrations. For *in vitro* experiments, a T_3 stock solution was prepared in dimethyl sulfoxide (DMSO) and further diluted in phosphate buffered saline (PBS). For whole cell electrophysiology studies (*Study III*) stock solutions of drugs were first dissolved in distilled water, except for CNQX and MK-801 that were first diluted in DMSO, and then diluted in artificial cerebral-spinal fluid (aCSF) at the desired final concentrations. In all *in vitro* experiments, final concentration of DMSO did not exceed 0.01%.

Thyroid hormones effects after experimental stroke (Study I)

For this study, 117 C57BL/6 male mice (20 to 26 g, aged nine to ten weeks, purchased from Charles River) were used. Out of 117 animals, 12 were excluded due to problems during surgery and mortality before entering the treatment phase and 105 animals were randomly assigned into the treatment groups (Fig. 1). Treatment was initiated on day two after photothrombosis (PT) and every other day until the endpoint of the study. Vehicle (Vh, NaCl 0.9%), T_3 (5 or 50 µg/kg) or T_4 (5 or 50 µg/kg) were administered by intraperitoneal injection in a total of six administrations. On days two, seven and 14 after stroke onset or sham surgery, animals were evaluated for motor function.

Photothrombosis. Focal ischemic stroke was induced by PT, as described previously [11, 16]. Briefly, animals were anesthetized with isoflurane in N_2O / O_2 (0.7: 0.3, 5% induction and 1.5 - 2 % maintenance) and placed into a stereotactic frame. After local anesthesia (bupivacaine 0.25 mg/ml), a sagittal skin incision was made on the scalp, subcutaneous connective tissue was removed, and the skull bone was dried. Five minutes after intraperitoneal injection (i.p.) of 1% (w/v) solution of the photosensitizing dye Rose Bengal (0.15 ml/mouse; Sigma-Aldrich, Taufkirchen, Germany) in NaCl 0.9%, the right hemisphere was illuminated with a cold light source (Schott KL 1500 LCD, intensity: 3200 K/5D) through an squared aperture measuring 4.0 to 2.0 mm (equal to an illumination area of 8.0 mm^2) for 20 minutes. The light position related to bregma (+1.5 mm lateral and +0.5 mm anterior) affected the mouse primary motor cortex of forelimb-responsive sites, in the left body side [13]. The same procedure was performed in Sham operated animals, with saline injection instead of photosensitizing dye. During anesthesia, the body temperature was monitored by a rectal probe (Linton Instrumentation, Norfolk, UK) connected to a heating pad maintaining body temperature at 36 - 37°C.

Behavior analysis. Motor function and exploratory behavior after thyroid hormones treatment was assessed using a neuroscore consisting of the rotating pole test (RPT) and the open field test, respectively [8, 15]. These assessments were performed in a blinded fashion to the investigator that performed the surgeries and treatments.

Rotating pole test. The RPT was used to assess postural and locomotor asymmetry that results from an unilateral brain lesion [4]. In brief, mice traversed a rotating wooden pole (length 1500 mm, diameter 40 mm, and elevation 700 mm) at zero, three, and ten rotations

per minute (rpm), to the right and left sides. Every animal was trained during three days before surgery and tested the day before PT. After stroke or sham surgery, animals were evaluated on day two for randomization into treatment groups. Each trial was video recorded, and videos were used to assess sensorimotor dysfunction by using a zero to six scoring system (Table 1). Animals that did not perform the behaviour test before the surgery (total score RPT < 20 points) or did not have motor deficits two days after PT (total score RPT > 15 points), were excluded from behaviour analysis. Behavioural analysis was performed in a blinded fashion to the investigator. In total, 42 animals were excluded from behaviour analysis and the following included: PT/Vh, n = 11; PT/T_3 5 μg/kg, n = 10; PT/T_3 50 μg/kg, n = 11; PT/T_4 5 μg/kg, n = 10; PT/T_4 50 μg/kg, n = 9.

Open field. The open field test was performed 14 days after stroke to assess both spontaneous post-ischemic locomotor activity and post-ischemic exploration behaviour [14]. Briefly, mice where placed into a square arena (44.5 cm × 44.5 cm) surrounded by 44.5 cm high sidewalls. The mouse was always placed in the center of the box and locomotion was recorded and the total distance traveled measured for five minutes.

Thyroid hormone levels determination. Animals were anesthetized with pentobarbital and plasma was collected 14 days after experimental stroke. Blood was collected from the heart into heparinized syringes and maintained at 2-8 ºC while handling. Plasma was collected after centrifugation 2000 x g for 10 minutes at 4 ºC and further stored at -80 ºC for further analysis. ELISA kit assay was used to determine TH levels in the plasma of mice. Plasma levels of T_3 and T_4 were determined by a commercial ELISA kits (ThermoFisher Scientific cat #EIAT3C and cat #EIAT4C, respectively) according to manufacturers instructions. In brief, plasma samples were incubated with specific primary antibodies in donkey anti sheep or goat anti mouse coated 96-well plates. After washing in respective buffer, plates were incubated with 3,3′,5,5′-tetramethylbenzidine substrate and absorbance was measured at 450 nm.

Immunohistochemistry and Immunofluorescence. Tissue collection for immunostainings was performed as described before [3, 9]. Fourteen days after PT animals were deeply anesthetized with pentobarbital and perfused fixed with paraformaldehyde (PFA) 4% and brains collected for immunohistochemistry analysis. Brain sagittal sections (thickness 30 μm) were washed three times in PBS and quenched with 3% H_2O_2 for

20 minutes. Brain sections were blocked for one hour at room temperature (RT), with 5% normal donkey serum (Jackson Immunoresearch, UK) in PBS supplemented with 0.25% Triton X-100. Sections were incubated with primary antibodies at 4 °C overnight, diluted in blocking solution.

Subsequently, slices were incubated with appropriate secondary biotinylated antibodies (donkey anti-rabbit / goat, Vector Laboratories, USA; 1:400) during 90 minutes at RT. Visualization was achieved through the Vectorstain ABC Elite kit (Vector Laboratories, CA, USA), 3,3'-diaminobenzidine tetrahydrochloride (DAB, DabSafe, Saveen Werner, Sweden), 8% $NiCl_2$ and 3% H_2O_2. Bright-field pictures were acquired using an Olympus BX60 microscope (Solna, Sweden), under standard conditions.

For immunofluorescence, brain sections were blocked as described above and incubated with primary antibodies at 4 ºC overnight. Primary antibodies used for immunofluorescence were rabbit TRβ1 (Millipore, 1:1000), rabbit TRα1 (Abcam, 1:1000), goat parvalbumin (PV235, Swant, 1:5000), mouse neuronal nuclei (NeuN, Millipore, 1:1000), glial fibrillary acidic protein (GFAP-Cy3; Sigma, 1:5000), rat CD68 (Abd Serotec, 1:300), and mouse GST-pi (BD Transduction Laboratories, 1:1000). The next day sections were incubated with appropriate secondary antibodies (donkey anti-rabbit/mouse/rat, 1:400) conjugated with fluorescent dyes Cy3, Cy5 (Jackson Immunoresearch, UK) or Alexa 488 (molecular Probes, Invitrogen, USA) for 90 minutes at RT. Images were acquired using an LSM510 confocal laser scanning fluorescence microscope (Carl Zeiss).

Infarct size measurement. Coronal brain sections from the start until the end of the infarct and spaced one millimeter were collected and stained for NeuN (rabbit NeuN, Millipore, 1:5000). Brain slices were mounted in Pertex and digitalized (CanoScan 8800F, Canon, Tokyo, Japan). The non-injured portion of the ipsilateral and contralateral hemisphere were encircled and the indirect infarct volume was calculated by integration of areas from serial sections of each brain as described previously [12], using Fiji software [10].

Counting of parvalbumin positive cells. For each animal one coronal section (-2.0 mm relative to bregma) was stained for Parvalbumin-positive (PV^+) neurons using a monoclonal goat primary antibody (PV235, Swant, 1:5000), and visualization accessed using a VECTOR NovaRED Peroxidase (HRP) Substrate Kit (Vector Laboratories, CA, USA). Rabbit c-fos (Santa Cruz, 1:500) positive immunoreactivity (c-fos^+) was accessed using the avidin–biotin–HRP system, as described before.

The following animals were included in this analysis: PT/Vh, n = 7; PT/T_3 50 µg/kg, n = 6; and PT/T_4 50 µg/kg, n = 4. Bright field images were acquired with 4x magnification objective and Fiji software was used to draw regions of interest, using an optical grid to define the distances and draw the regions. PV^+ cells and PV^+/c-fos^+ in the peri-infarct somatosensory cortex (area of 0.8 mm^2) and homotypic area in the contralateral hemisphere were counted. The infarct core was identified by the lack of NeuN immunoreactivity in subsequent sections.

Immunoblotting. Brains from mice were collected as previously described [9]. Fourteen days after PT animals were deeply anesthetized with pentobarbital and brains were immediately frozen (−40°C) in isopentane (Sigma-Aldrich, Taufkirchen, Germany) and further cooled down to −70°C on dry ice for immunoblotting. Fresh frozen brains were placed into a brain matrix and cut (+2.2 mm to -2.2 mm relatively to bregma). For each four millimeters thick section, the tissue correspondent to the infarct core and peri-infarct was collected. The procedure was performed in a refrigerated chamber at -20°C.

Tissue from human brains were dissected out by a pathologist following autopsy. Brain tissues were immediately frozen and stored at -80 ºC, temporarily moved to a refrigerated chamber at -20 ºC to excise small specimens and stored at -80 ºC until protein extraction.

Western blot. Proteins were dissected from brain tissue as described before [2, 9]. Briefly, brain tissue was mechanically homogenized by a Dounce homogenizer in lysis buffer containing 20 mM Tris (pH 7.5), 150 mM NaCl, 1 mM EDTA, 1 mM EGTA, 2.5 mM sodium pyrophosphate, 1 mM β-glycerolphosphate, 1 mM sodium orthovanadate (Na_3VO_4), 1 mM phenylmethanesulfonyl fluoride (PMSF), 1% triton-X 100 and supplemented with protease inhibitor cocktail (Sigma-Aldrich, Deisenhofen, Germany). The homogenates were centrifuged 14.000g at 4°C for 20 minutes, after 20 minutes of incubation on ice. The supernatant was collected and stored at -20°C for further analysis. Whole protein concentrations were determined by the Bradford method using bovine serum albumin (BSA, Sigma-Aldrich) in lysis buffer as standard.

Brain lysates were diluted in sodium dodecyl sulfate (SDS) sample buffer and proteins denatured at 94 °C for five minutes. Five or ten micrograms of protein were separated on a 10% or 15% SDS polyacrylamide gel. After transferring proteins onto polyvinylidene difluoride membranes, these were blocked with 5% non-fat dry milk solution in tris buffered saline with Tween 20 1% (TBS-T). Following blocking, membranes were incubated with primary antibody in 5% BSA solution in TBS-T, at 4 °C overnight. Primary antibodies used for western blot were rabbit TRα1 (Abcam, 1:1000), rabbit TRβ1 (Millipore, 1:20000), mouse

postsynaptic protein 95 (PSD95; BD Transduction Laboratories, 1:1000), rabbit synaptophysin (Thermoscientific, 1:15000), rabbit glutamate receptor 1 (GluR1; Millipore, 1:2000), mouse GluR2 (Millipore, 1:1000), mouse *N*-methyl-D-aspartate receptor 1 (NMDAR1; BD Transduction Laboratories, 1:1000), rabbit synaptotagmin 1&2 (Abcam, 1:1000) and rabbit glutamic acid decarboxylase 65/67 (GAD 65/67; Millipore, 1:2000). After blocking, the membranes were incubated with a rabbit / mouse secondary HRP-conjugated antibody (Sigma-Aldrich, Stockholm, Sweden, 1:15000 / 1:10000, respectively) for one hour at RT. The signals were visualized by using a chemiluminescence kit (Merck Millipore, Germany) and CCD camera (Fujifilm LAS 1000, Fujifilm, Tokyo, Japan). Membranes were reprobed with anti β-actin HRP conjugated (1:150000, Sigma-Aldrich). Levels were calculated as a percentage of β-actin expression, after densitometric analysis using Fiji software.

Dynamics of dendritic spines after administration with T_3 (Study II)

To study the effects of T_3 on dendritic spine dynamics in mouse neocortical neurons after experimental stroke, eight Thy1- yellow fluorescent protein (YFP) transgenic mice (25 to 40 g, aged one year, own breeding), that express YFP in neuronal population were used. Mice were randomly assigned in the following treatment groups: PT/Vh, n = 4; PT/T_3 50 μg/kg, n = 4 (Fig. 1). Treatment was administered as described above for *Study I*. Fourteen days after the surgery, mice were sacrificed, perfusion fixed with PFA 4% and brains were collected for further infarct volume assessment and dendritic spine analysis.

Photothrombosis. To induce PT in animals for dendritic spine analysis (*Study II*) the surgical procedure as described for *Study I*, and the left hemisphere was illuminated with a cold light source (Schott KL 1500 LCD, intensity: 3050 K/4D) through a round aperture measuring 1.5 mm in diameter (equal to an illumination area of 1.767 mm^2) for 20 minutes. This approach induced smaller infarct sizes so that dendritic spines could be analyzed in different regions in the peri-infarct area. The same procedure was performed in Sham operated animals, with saline injection instead of photosensitizing dye.

Detection and classification of dendritic spines from fluorescence Laser Scanning Microscopy. Brain coronal sections (thickness 30 μm) were rinsed in PBS, mounted on super charged slides, and cover-slipped with PVA-DABCO (Sigma-Aldrich). Images were acquired using an AxioObserver LSM 710 confocal (Carl Zeiss) using a Plan-Apochromat 63x/1.4 DIC M27 oil immersion objective (Carl Zeiss, Jena, Germany) and ZEN 2010 imaging software.

Three coronal sections per animal were collected at different levels: +2.0 mm, +1.0 mm and 0 mm relatively to bregma, corresponding to the rostral pole, center and caudal pole of the infarct, respectively. For each animal, we analyzed layers II/III correspondent to the apical pyramidal neurons in the ipsilateral motor cortex (Region 1, R1), ipsilateral somatosensory cortex (Region 2, R2), contralateral motor cortex (Region 3, R3) and contralateral somatosensory cortex (Region 4, R4).

Images were obtained in z-stack planes of 224.8 × 224.8 μm in *xy* and 15 to 16 μm in *z* (*x/y/z*, 0.11 pixel/μm), resolution 2048 x 2048 pixels in *xy* and spaced 0.2 μm in *z*.

Dendritic spine density and shape classification was accurately quantified and characterized using a three dimensional (3D) computational approach as previously described [5]. After median filter application to reduce noise, radius of 1.0 pixel, we performed proper image 3D deconvolution using interactive deconvolve 3D plugin from Fiji software, after theoretical point spread function (PSF) generation using diffraction PSF 3D plugin. Deconvolution restores image contrast that is lost during image recording due to the optical smearing introduced by the PSF of the microscope, and it is an important systematic error correction for dendritic spine analysis by improvement of signal to noise ratio [5].

For each region, three to five dendritic branches were randomly selected. Dendrites were manually selected, and spines were automatically detected using NeuronStudio software. Minimum and maximum height for spines were set to 0.2 μm and 2.0 μm, respectively, and voxel dimensions were adjusted for our images (0.098 μm, 0.098 μm and 0.2 μm for *xyz*, respectively). For spine shape classification, we used the Rayburst algorithm provided by NeuronStudio software [5, 6], which allowed to use all the information from a LSM image stack, and also provided procedures as declumping of merged spines and spine stem reattachment, making spine detection more accurate than manual or 2D method, where spines are masked along *z* axis. Dendritic spines were classified according to head to neck ratio and head diameter as stubby, mushroom or thin [1, 5], using default parameters from NeuronStudio. Some detected dendritic spines from neighbor dendritic branches were manually deleted and not included in statistical analysis. Dendritic spine density was calculated with the ratio number of spines / dendrite length.

In vitro modulation of T_3 in glutamatergic neurons (Study III)

An *in vitro* model of cerebral ischemia and electrophysiology studies were performed to study immediate effects of T_3 in homeostatic plastic mechanisms, namely modulation of synaptic proteins crucial for neurotransmission and ionotropic glutamate receptors alpha-amino-3-hydroxy-5-methyl-4-isoxazolepropionic acid (AMPA) and *N*-methyl-D-aspartate (NMDA) evoked currents.

Cell cultures. *C*ultured glutamatergic cortical neurons were used after 7 - 8 days *in vitro* (DIV). Primary cortical neuronal cultures were prepared as described before [7]. Cells were obtained from the cerebral cortex from Wistar rats on embryonic day 16 - 18. Briefly, meninges were removed, and the cortex dissected and subjected to enzymatic dissociation, using 0.05 / 0.02% w/v in PBS trypsin / EDTA (#15400054, Thermofisher) for 15 minutes at 37 ºC. The homogenized was rinsed with Dulbecco's Modified Eagle's medium (#11880036, DMEM, GIBCO) with 10% fetal bovine serum (#10500-064, GIBCO), 100 U penicillin and streptomycin/ml (#15140122, Thermofisher), 2 mM L-glutamine (#G5792, Sigma-Aldrich), dissociated with a Pasteur pipette, centrifuged and redissociated in starter medium (#21103049, Neurobasal medium, GIBCO) supplemented with B27 (#17504044, GIBCO), 100 U penicillin and streptomycin/ml, 2 mM L-glutamine (#G5792, Sigma-Aldrich) and 25 µM glutamate (#49621, Sigma-Aldrich). The cells were plated onto poly-L-lysine (#P4707, Sigma-Aldrich) pre-coated multiwells at 1.5×10^5 cells/cm^2 and grown in starter medium at 37 ºC and 5% CO_2. One-half of the medium was replaced with cultivating medium (starter medium without glutamate) from 4 DIV. Cells were used after 7-8 DIV for *in vitro* assays.

In vitro ischemic model and experimental treatments. After 7 DIV, neurobasal medium was collected and stored to be replaced after experiments. Neuron cultures were washed with PBS, and oxygen and glucose deprivation (OGD) was induced with deoxygenated aglycemic solution (in mM): 143.8 Na^+, 5.5 K^+, 1.8 Ca^{2+}, 1.8 Mg^{2+}, 125.3 Cl^-, 26.2 HCO^{3-}, 1.0 PO_4^{3-}, 0.8 SO_4^{2-}, pH 7.4) in an anoxic atmosphere. OGD was generated in a hypoxia incubator chamber (StemCell Technologies), flushed with gas: 5% CO_2, 95% N_2. In control cultures, medium was replaced by basic salt solution (BSS) after washing with PBS (in mM): 143.8 Na^+, 5.5 K^+, 1.8 Ca^{2+}, 1.8 Mg^{2+}, 125.3 Cl^-, 26.2 HCO^{3-}, 1.0 PO_4^{3-}, 0.8 SO_4^{2-}, 20 glucose, pH 7.4), and cells were incubated in a normoxic atmosphere containing 5% CO_2. Cultures were in OGD or BSS solutions for 120 minutes and after replaced by previous collected medium. After OGD / BSS conditions, cells were incubated with Vh (DMSO in

PBS, 0.01%) or T_3 1 µM for 48 hours. Subsequently, cells were washed with cold PBS to remove excess of culture medium and cells collected and frozen at -80 ºC until protein extraction.

Immunocytochemistry. For immunocytochemistry, neurons were plated on glass coverslips and fixed in PFA 4% for 10 min after 7 DIV. Immunofluorescence was performed as described for brain sections, and coverslips were incubated overnight with rabbit TRα1 (Thermoscientific, 1:500) or rabbit TRβ1 (Millipore, 1.500). The next day, neurons were incubated with secondary biotinylated antibody donkey anti-rabbit, 1:400 (Jackson Immunoresearch, UK) for 90 minutes at RT and Streptavidin Alexa 488 (molecular Probes, Invitrogen, USA) for 60 minutes at RT. Next, glass coverslips were stained with Hoechst-33342 (4 µg/ml, Life Technologies) for 5 min at RT and mounted in Dako fluorescent mounting medium. Photomicrographs were obtained using an AxioObserver LSM710 confocal microscope (Carl Zeiss).

Immunobloting. Protein extraction was performed as previously described [2, 9]. Western blots were performed as described above for brain extracts, and mouse synaptotagmin (BD Transduction Laboratories, 1:2000) was incubated overnight at 4 ºC. After blocking, the membranes were incubated with a mouse secondary HRP-conjugated antibody (Sigma-Aldrich, Stockholm, Sweden, 1:10000) for one hour at RT.

Electrophysiological recording of membrane currents. To study ligand-gated channels AMPA and NMDA, we adopted the voltage-ramp method [17]. Currents were amplified with an Axopatch 200B (Axon Instruments, USA) and digitized at a frequency of 10 kHz and filtered at 0.1 kHz using the analog-to-digital converter Digidata 1322A (Axon Instruments, USA) and pClamp software (version 8, Axon Instruments, USA). During recording, cells were maintained at RT (21 – 25 ºC) in aCSF filtered through a 0.45 µm mesh, of the following composition (in mM): 126 NaCl, 3 KCl, 2 $MgSO_4$, 2 $CaCl_2$, 1.25 NaH_2PO_4, 10 glucose and 10 HEPES, 290 mOsmol. The internal solution of the recording electrode was composed of (in mM) 115 K-gluconate, 4 NaCl, 0.3 GTP and 2 ATP-Mg, pH 7.2 with KOH, 270 mOsmol.

Electrodes were pulled on a vertical puller (PC-10, Narishige), from borosilicate glass capillaries (Harvard Apparatus). The initial patch microelectrode had a resistance of 5.6 –

7.4 MΩ, when filled with internal solution. The electrode was sealed against cells at least 1.0 GΩ, and membrane was ruptured by suction pulses, which allows the recording of the intracellular membrane potential. Only cells with Ra (Access resistance) values < 10 MΩ were included.

Solutions were delivered diluted in the bath solution through a custom-made perfusion system, where capillary tubes with 250 µm inner diameter merged into a common outlet. Drugs were applied close to cell at approximately 50 to 100 µm, at a rate of 20 µl/min.

Individual currents were recorded in the presence of T_3 1 µM (n = 4) or Vh (n = 3), that were incubated 48 hours before the experiments. A sequence of voltage ramps at a rate of 0.23 mV/millisecond were applied at a holding potential of -80 mV. To obtain the agonist induced current-voltage (I-V) relation, ramps I-V curves were constructed applying a 500 milliseconds voltage ramp ranging from -110 mV to +20 mV elicited every 8 seconds. Voltage ramps were applied in the absence and in the presence of AMPA and NMDA agonist glutamate at 50 µM and co-agonist of NMDA channels glycine at 3 µM, to enable subtraction of leak currents. CNQX and MK-801 were used both at 10 µM as antagonists of AMPA and NMDA channels, respectively. For stabilization of background currents, a minimum of 80 seconds was recorded before agonists and CNQX / MK-801 application.

Cell currents were recorded sequentially in the presence of specific K^+- channel blockers tetraethylammonium sodium salt (5 mM) and 4-Aminopyridine (1 mM), that were applied in the perfusion system together with the other drugs. Voltage-gated K^+ channels needed to be blocked, since those channels were contributing to the conductance as well to the reversal potential obtained.

References

1. Harris KM, Jensen FE, Tsao B (1992) Three-dimensional structure of dendritic spines and synapses in rat hippocampus (CA1) at postnatal day 15 and adult ages: implications for the maturation of synaptic physiology and long-term potentiation. J Neurosci 12:2685–2705

2. Kuric E, Ruscher K (2014) Reduction of rat brain CD8+ T-cells by levodopa/benserazide treatment after experimental stroke. Eur J Neurosci 40:2463–2470. doi: 10.1111/ejn.12598

3. Quattromani MJ, Cordeau P, Ruscher K, Kriz J, Wieloch T (2014) Enriched housing down-regulates the Toll-like receptor 2 response in the mouse brain after experimental stroke. Neurobiol Dis 66:66–73. doi: 10.1016/j.nbd.2014.02.010

4. Rickhag M, Deierborg T, Patel S, Ruscher K, Wieloch T (2008) Apolipoprotein D is elevated in oligodendrocytes in the peri-infarct region after experimental stroke: Influence of enriched environment. J Cereb Blood Flow Metab 28:551–562. doi: 10.1038/sj.jcbfm.9600552

5. Rodriguez A, Ehlenberger DB, Dickstein DL, Hof PR, Susan L (2008) Automated Three-Dimensional Detection and Shape Classification of Dendritic Spines from Fluorescence Microscopy Images. 3:e1997. doi: 10.1371/journal.pone.0001997

6. Rodriguez A, Ehlenberger DB, Hof PR, Wearne SL (2006) Rayburst Sampling, an Algorithm for Automated Three-dimensional Shape Analysis from Laser Scanning Microscopy Images. Nat Protoc 1:2152–2161. doi: 10.1038/nprot.2006.313

7. Ruscher K, Freyer D, Karsch M, Isaev N, Megow D, Sawitzki B, Priller J, Dirnagl U, Meisel A (2002) Erythropoietin Is a Paracrine Mediator of Ischemic Tolerance in the Brain: Evidence from an In Vitro Model. J Neurosci 22:10291–10301. doi: 10.1523/jneurosci.22-23-10291.2002

8. Ruscher K, Johannesson E, Brugiere E, Erickson A, Rickhag M, Wieloch T (2009) Enriched environment reduces apolipoprotein E (ApoE) in reactive astrocytes and attenuates inflammation of the peri-infarct tissue after experimental stroke. J Cereb Blood Flow Metab 29:1796–1805. doi: 10.1038/jcbfm.2009.96

9. Saraiva C, Talhada D, Rai A, Ferreira R, Ferreira L, Bernardino L, Ruscher K (2018) MicroRNA-124-loaded nanoparticles increase survival and neuronal differentiation of neural stem cells in vitro but do not contribute to stroke outcome in vivo. PLoS One 13:e0193609. doi: 10.1371/journal.pone.0193609

10. Schindelin J, Arganda-Carreras I, Frise E, Kaynig V, Longair M, Pietzsch T, Preibisch S, Rueden C, Saalfeld S, Schmid B, Tinevez JY, White DJ, Hartenstein V, Eliceiri K, Tomancak P, Cardona A (2012) Fiji: An open-source platform for biological-image analysis. Nat. Methods 9:676–682

11. Schroeter M, Jander S, Stoll G (2002) Non-invasive induction of focal cerebral ischemia in mice by photothrombosis of cortical microvessels: Characterization of inflammatory responses. J Neurosci Methods 117:43–49. doi: 10.1016/S0165-0270(02)00072-9

12. Swanson RA, Morton MT, Tsao-Wu G, Savalos RA, Davidson C, Sharp FR (1990) A semiautomated method for measuring brain infarct volume. J Cereb Blood Flow Metab 10:290–293. doi: 10.1038/jcbfm.1990.47

13. Tennant KA, Adkins DL, Donlan NA, Asay AL, Thomas N, Kleim JA, Jones TA (2011) The organization of the forelimb representation of the C57BL/6 mouse motor cortex as defined by intracortical microstimulation and cytoarchitecture. Cereb Cortex 21:865–876. doi: 10.1093/cercor/bhq159

14. Walsh RN, Cummins R a (1976) The Open-Field Test: a critical review. Psychol Bull 83:482–504. doi: 10.1037/0033-2909.83.3.482

15. Walter HL, van der Maten G, Antunes AR, Wieloch T, Ruscher K (2015) Treatment with AMD3100 attenuates the microglial response and improves outcome after experimental stroke. J Neuroinflammation 12:24. doi: 10.1186/s12974-014-0232-1

16. Watson BD, Dietrich WD, Busto R, Wachtel MS, Ginsberg MD (1985) Induction of reproducible brain infarction by photochemically initiated thrombosis. Ann Neurol 17:497–504. doi: 10.1002/ana.410170513

17. Yelhekar TD, Druzin M, Karlsson U, Blomqvist E, Johansson S (2016) How to Properly Measure a Current-Voltage Relation?—Interpolation vs. Ramp Methods Applied to Studies of GABAA Receptors. Front Cell Neurosci 10:10. doi: 10.3389/fncel.2016.00010
